# Supplementary material for: A cross-species assessment of behavioral flexibility in compulsive disorders
Source: Commun Biol. 2021 Jan 21;4:96. doi: 10.1038/s42003-020-01611-y (PMC7820021; doi:10.1038/s42003-020-01611-y)
Supplement: Supplementary file 1 — Supplementary Information [file 42003_2020_1611_MOESM1_ESM.pdf]

Supplementary information for:

A cross-species assessment of behavioural flexibility in compulsive disorders

Nabil Benzina[1], Karim N'Diaye[1], Antoine Pelissolo[2, 3], Luc Mallet[1, 2, 4], Eric Burguière[1]

1. Institut du Cerveau, ICM, Inserm U 1127, CNRS UMR 7225, Sorbonne Université
47 bd de l'Hôpital, 75013 Paris, France
2. Assistance Publique-Hôpitaux de Paris, DMU IMPACT, Département Médical-Universitaire de Psychiatrie et d'Addictologie, Hôpitaux Universitaires Henri Mondor - Albert Chenevier, Université Paris-Est Créteil
40 rue de Mesly, 94000 Créteil, France
3. INSERM U955, IMRB
8 rue du Général Sarrail, 94010 Créteil cedex, France
4. Department of Mental Health and Psychiatry, Global Health Institute, University of Geneva
9 Chemin des Mines , 1202 Geneva, Switzerland

Correspondence should be addressed to Nabil Benzina and Eric Burguière, Institut du Cerveau, Groupe Hospitalier Pitié-Salpêtrière, 47-83, boulevard de l'Hôpital, 75013 Paris, France; Phone: +33 1 57 27 44 25 ; E-mail: nabil.benzina@unige.ch ; eric.burguiere@icm-institute.org

Supplementary Method

Human reversal learning task: additional behavioural parameters

In addition to the main behavioural parameters (number of trials needed to reach the reversal criterion, reversal errors, SSC probability, SSC errors), two additional parameters compared to mice were also extracted: the probability of a strategy change after a probabilistic error (SCAPE, i.e., switching to the unrewarded stimulus following misleading negative feedback to a correct response) and the number of perseverative errors following a SCAPE (SCAPE errors).

Mouse instrumental pre-training

Prior to the beginning of the task, the mice were first habituated to the experimental chamber and the food for 24 hours with a pellet delivered each time they nose poked in the pellet receptacle (two deliveries being separated by at least 5 minutes). After this habituation phase, they underwent automatically two phases of instrumental pre-training. In the first phase, the mouse had to learn to touch any of the screen (which were off with no visual stimulus) in order to get a pellet as a reward. The two screens blinked during 15 s after each touch to indicate the availability of a reward. The reward was delivered even if the mouse was not retrieving the reward within this 15 s time period. If the mouse succeeded to do 10 consecutive reward retrievals within 15 s, the second phase was automatically initiated. In the second phase, the mouse had to learn to initiate a trial by doing a nose poke in the pellet receptacle. When they did so, the screens turned on white for 60 s, indicating the mouse to touch one of them. If it did so before the screens turned off, the screen blinked for 15 s as a signal to retrieve the reward. After 10 consecutive successful trials of this second pre-training phase, the reversal learning task was automatically initiated.

Two-step cluster analysis

As its name suggests it, this algorithm is based on a two-stage approach: in the first stage, the algorithm undertakes a procedure that is very similar to the k-means algorithm. Based on these results, the procedure conducts a modified hierarchical agglomerative clustering procedure that combines the objects sequentially to form homogenous clusters. This algorithm has the advantage to automatically choose the number of clusters to retain by calculating measures of fit such as the Bayes Information Criterion (BIC).

The silhouette measure of cohesion and separation is essentially based on the average distances between the objects and can vary between -1 and +1.

Supplementary Notes

Cluster analysis in OCD patients

We performed the same cluster analysis with the same behavioural parameters as for mice in order to confirm the relevance of a subgroup split based on the clinical dimension. This analysis revealed an impaired subgroup of 7 patients representing 17.5% of all our patients; 85.7% of them in this subgroup being checkers (6 out of 7 patients). The model with 2 patients sub-groups differing in their performance in our task was automatically retain with a BIC of 137.72 (142.09 for no sub-population and 154.03 for 3 sub-populations) and a ΔBIC of -4.37 indicating a positive evidence[1] in favour of it (a ΔBIC between 0 and 2 corresponding to a weak evidence; 2 and 6, a positive evidence; 6 and 10, a strong evidence; and more than 10 a very strong one). The silhouette measure was 0.6, indicating a good solution. The same analysis was applied to healthy subjects but no cluster was found. As for *Sapap3* KO mice, the SSC probability was the most important variable for clusters identification (importance value of 1), followed by the number of trials needed to reach reversal criterion (0.53), the SSC perseverative errors (0.36), and the number of reversal errors (0 therefore no importance). The fact that the composition of this subgroup was almost exclusively checkers reinforces the relevance of using this clinical dimension, as we did as a first intention, to study the different OCD subgroups in the context of behavioural flexibility.

No influence of medication status on task performance in OCD patients

To confirm the absence of influence of antidepressant treatment on task performance, we separated patients into two subgroups according to their treatment status (medicated (n = 28) vs. unmedicated (n = 12) patients) and compared their performance. Thus, no difference between the groups emerged whether for the number of trials needed to reach the reversal criterion (BF_{10} = 0.51, *d* = -0.28 [-0.91 0.31]), or for the reversal errors (BF_{10} = 0.33, *d* = 0.05 [-0.54 0.64]), the SSC probability (BF_{10} = 0.61, *d* = -0.33 [-0.98 0.26]) and the SSC errors (BF_{10} = 0.33, *d* = -0.01 [-0.6 0.58]).

The higher response lability is reversal specific

In order to determine if the increased response lability is reversal specific, we compared the SSC probability in the acquisition phase for both species. In humans, we did not find a group effect (BF_{10} = 0.41, η^2 = 0.04). In mice, a group effect seemed to exist (BF_{10} = 1.55, η^2 = 0.12) with no difference between “impaired” KO mice and WT controls (43 ± 4.5% vs 45.54 ± 8.74%, BF_{10} = 0.47, *d* = 0.33 [-0.34 0.88]) and between “impaired” and “unimpaired” KO mice (43 ± 4.5% vs 39.3 ± 6.84%, BF_{10} = 0.92, *d* = -0.63 [-1.24 0.2]) while “unimpaired” KO mice tend to be less labile than WT controls (BF_{10} = 2.44, *d* = 0.77 [0.03 1.31]). Thus, both OCD “checkers” and “impaired” KO mice seem to show response lability only in a reversal context.

Supplementary Discussion

Although more complex tasks were developed to study cognitive flexibility to a higher degree in humans[2], we chose a reversal learning task in order to measure the same construct in both species. Indeed, the various components of cognitive flexibility are based on various regions of the prefrontal cortex (PFC)[3]. However, there are strong divergences between a human and a rodent PFC[4–7]. Thus, this task was of particular interest for its ability to measure the simplest and most conserved form of cognitive flexibility through species, namely the ability to reverse a stimulus-reward association[8]. Moreover, it is essentially supported for both humans and mice by the OFC[9–11] which is a PFC region shared by both species[12–15], the activity of which is altered in OCD patients whether at rest[16] or during the execution of this task[17]. We have also strengthened the cross-species validity of our task by excluding the paradigms classically used in rodents based on spatial discrimination. We rather developed a reversal learning task in mice based on visual discrimination, the most commonly used sensory modality in human studies. Some teams already tried to transpose humans' experimental paradigms to rodents using the same modalities[18–20]. However, they remain a minority with scarcely any studies assessing patients and animal models in parallel[21].

Since sight is a less important sense in rodents, some might argue that it would have been wiser to rely on a sensory modality naturally more developed in this species such as smell, to ensure that the impairment is not due to a difficulty in discriminating stimuli. However, beyond the fact that we used visual stimuli validated in rodents[22], the use of species-specific capabilities (as the use of olfactory stimuli) does not allow the generation of data that can be readily generalized to other species[23]. On the contrary, the further away from species-specific abilities/behaviours, the more likely it is to assess a function/mechanism that transcends the species barrier[24]. Another concern may rely on the use of deterministic feedbacks in rodents rather than probabilistic feedbacks. The choice of probabilistic feedbacks in humans was justified to increase the difficulty of the task as the use of deterministic feedback made it so simple that participants immediately detect the change in contingencies and start responding to the other stimulus. This issue does not arise in rodents for which the task is difficult enough in its deterministic version as it requires hundreds of trials to achieve only one reversal. It has also been shown that this difference is insignificant given the similarity of the data acquired in the two species using this paradigm[25]. Nevertheless, this difference could explain some difference observed in our results, such as the lower tendency to persevere of the "impaired" *Sapap3* KO mice compared to control, not observed in the human version of the task with OCD checkers. The difference between the two species could be explained by the fact that the human version of the task was probabilistic and thus a negative feedback could be interpreted as a probabilistic error and therefore not lead to a switch in their response; patients not having an increased sensitivity to probabilistic negative feedback.

In parallel to the effective implementation of a translational approach, one of the strengths of our study lies in the development of a setup dedicated to the behavioural assessment in naturalistic condition of our mice with a high throughput data acquisition allowing to obtain results unbiased by the stress induced by the environment or the experimenter[26–28]. Indeed, most of animal studies relies on food deprivation, multiple labour-intensive sessions with daily manipulations without any respect towards the animal physiological cycle causing

stress in animals. These factors are not fully considered with very few teams taking an interest in them and developing fully automated procedures that allow the animal to live and work in the experimental apparatus without these stress factors[29–31].

Supplementary tables

Model Comparison

Models	BF $_{10}$
Null model	1.000
Trial	3.251e +244
Trial + OCD	3.651e +243
Trial + OCD + Trial ✻ OCD	2.683e +243
OCD	0.094

Analysis of Effects

Effects	BF $_{Inclusion}$
Trial	∞
OCD	0.130
Trial ✻ OCD	0.297

Note. Compares models that contain the effect to equivalent models stripped of the effect. Higher-order interactions are excluded.

Supplementary Table S1. OCD did not influence the performance following a reversal event.

Model Comparison

Models	BF $_{10}$
Null model	1.000
Trial	4.616e +35
Trial + KO	1.028e +35
Trial + KO + Trial ✻ KO	4.600e +30
KO	0.215

Analysis of Effects

Effects	BF $_{Inclusion}$
Trial	∞
KO	0.149
Trial ✻ KO	3.260e -5

Note. Compares models that contain the effect to equivalent models stripped of the effect. Higher-order interactions are excluded.

Supplementary Table S2. The knockout of the *Sapap3* gene did not influence the performance following a reversal event.

Model Comparison

Models	BF $_{10}$
Null model	1.000
Checking subtype	3.975
Comorbid anxiety disorder	0.332
Checking subtype + Comorbid anxiety disorder	2.069
Checking subtype + Comorbid anxiety disorder + Checking subtype ✻ Comorbid anxiety disorder	1.250

Analysis of Effects

Effects	BF $_{Inclusion}$
Checking subtype	4.539
Comorbid anxiety disorder	0.483
Checking subtype ✻ Comorbid anxiety disorder	0.604

Note. Compares models that contain the effect to equivalent models stripped of the effect. Higher-order interactions are excluded.

Supplementary Table S3. No effect of having a comorbid anxiety disorder on the mean number of trials needed to reach reversal criterion.

Model Comparison

Models	BF $_{10}$
Null model	1.000
Checking subtype	3.975
Gender	0.411
Checking subtype + Gender	1.337
Checking subtype + Gender + Checking subtype ✻ Gender	0.361

Analysis of Effects

Effects	BF $_{Inclusion}$
Checking subtype	3.764
Gender	0.351
Checking subtype ✻ Gender	0.270

Note. Compares models that contain the effect to equivalent models stripped of the effect. Higher-order interactions are excluded.

Supplementary Table S4. No effect of gender on the mean number of trials needed to reach reversal criterion.

Model Comparison

Models	BF $_{10}$
Null model	1.000
Checking subtype	1.189
Comorbid anxiety disorder	0.361
Checking subtype + Comorbid anxiety disorder	0.444
Checking subtype + Comorbid anxiety disorder + Checking subtype ✻ Comorbid anxiety disorder	0.163

Analysis of Effects

Effects	BF $_{Inclusion}$
Checking subtype	1.200
Comorbid anxiety disorder	0.367
Checking subtype ✻ Comorbid anxiety disorder	0.367

Note. Compares models that contain the effect to equivalent models stripped of the effect. Higher-order interactions are excluded.

Supplementary Table S5. No effect of having a comorbid anxiety disorder on the probability of spontaneous strategy change.

Model Comparison

Models	BF $_{10}$
Null model	1.000
Checking subtype	1.189
Gender	0.303
Checking subtype + Gender	0.312
Checking subtype + Gender + Checking subtype ✻ Gender	0.250

Analysis of Effects

Effects	BF $_{Inclusion}$
Checking subtype	1.152
Gender	0.281
Checking subtype ✻ Gender	0.802

Note. Compares models that contain the effect to equivalent models stripped of the effect. Higher-order interactions are excluded.

Supplementary Table S6. No effect of gender on the probability of spontaneous strategy change.

Supplementary figures

Supplementary Figure S1

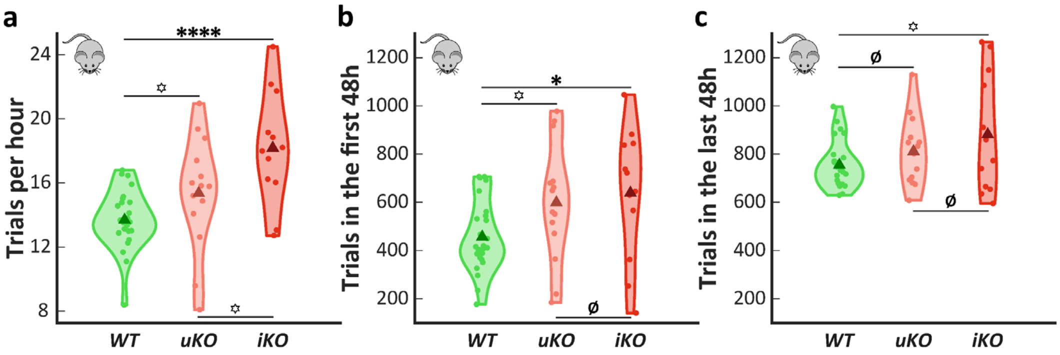

Supplementary Figure S2

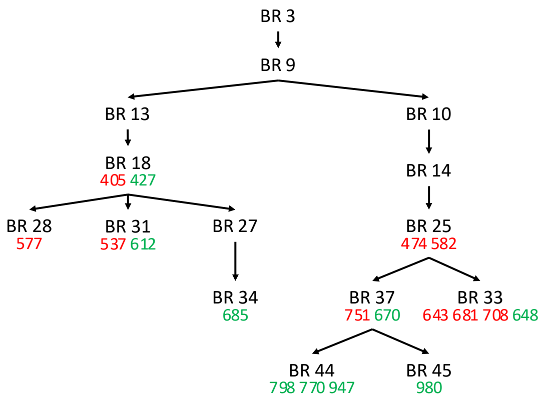

Supplementary Figure S3

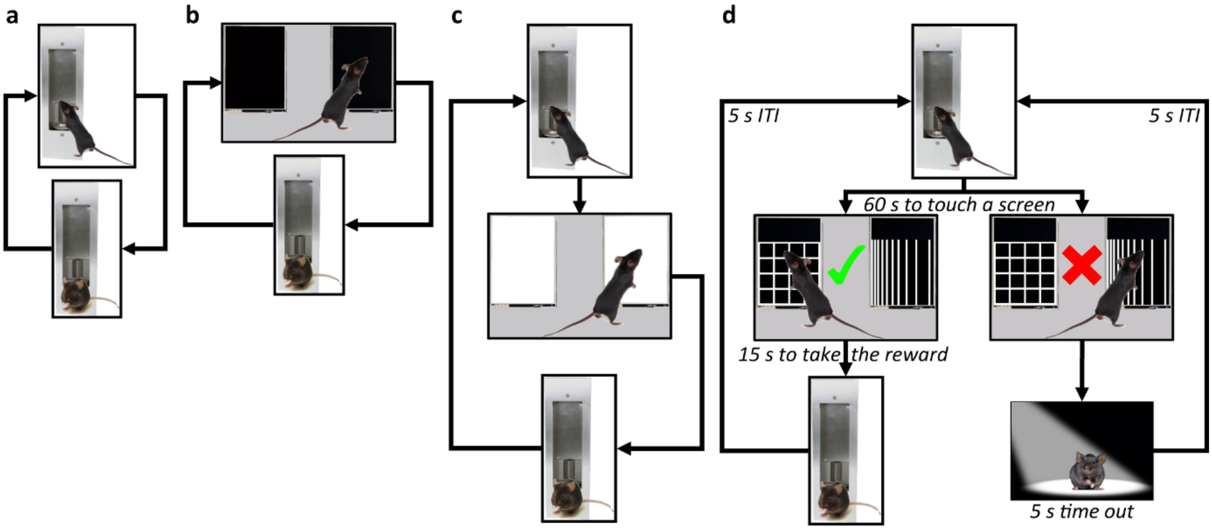

Supplementary Figure S4

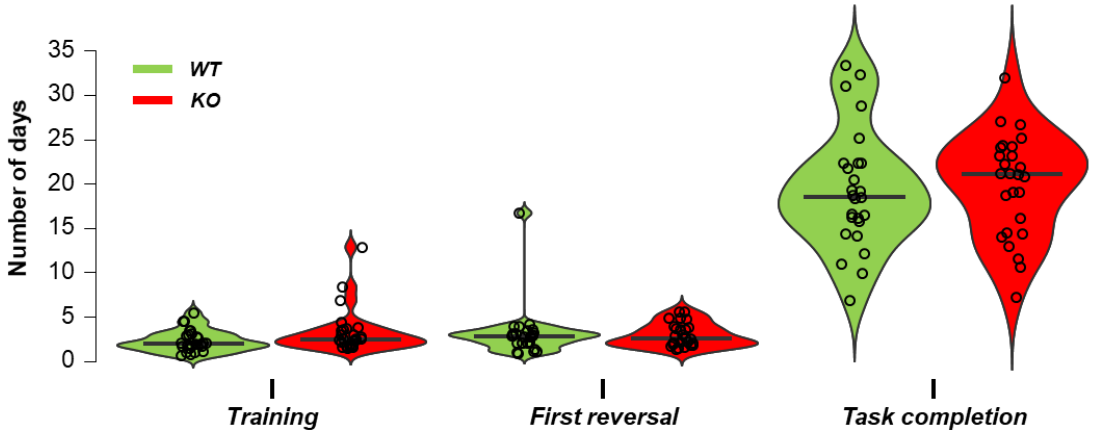

Supplementary figure legends

Supplementary Figure S1. The *Sapap3* KO mice are more active compared to WT mice. **(a)** The *Sapap3* KO mice performed more trials per hour in average than WT mice with the impaired KO mice subgroup having the highest rate. **(b)** The *Sapap3* KO mice performed more trials in the first 48 hours after the beginning of the task. **(c)** No significant difference in engagement level in the last 48 hours of the task (3 weeks after the beginning of the task in average). All mice were significantly more engaged in the last 48 hours compared to the first 48 hours (JZS two-way mixed ANOVA, $BF_{10} > 100$, $\eta^2 = 0.5$; with no group influence over time, $BF_{10} = 0.99$, $\eta^2 = 0.02$). *uKO: "unimpaired" KO mice. iKO: "impaired" KO mice. Triangle: group mean. Dot: individual mean.*
*ø: $BF_{10} < 1$. ✡: $BF_{10} < 3$. *: $BF_{10} \geq 3$. **: $BF_{10} \geq 10$. ***: $BF_{10} \geq 30$. ****: $BF_{10} \geq 100$.*

Supplementary Figure S2. *Sapap3* KO mice genealogy. *BR = breeding pair. In green: unimpaired KO mouse. In red: impaired KO mouse.*

Supplementary Figure S3. The four stages of the mouse version of the task. **(a)** Habituation to the experimental chamber and the food for 24 hours. *A pellet is delivered each time the mouse nose poke in the pellet receptacle (two deliveries being separated by at least 5 minutes).* **(b)** Screen touch learning: the mouse has to learn to touch the screens in order to get a pellet as a reward. *One screen touch initiates a 15-second blink to indicate the reward availability. This stage ends with 10 rewards consecutively retrieved within 15 seconds.* **(c)** Trial initiation learning: the mouse has to learn to launch a trial before touching a screen in order to get a reward. *A nose poke in the pellet receptacle turns on white the screens for 60 s, indicating the mouse to touch one of them. A screen touch within 60 s turns them off and launch a 15-second blink to indicate the reward availability. This stage ends with 10 rewards consecutively retrieved within 15 s.* **(d)** A trial sequence of the reversal stage. *First, the mouse has to nose poke into the pellet receptacle to launch a trial, triggering the stimuli display. It has then 60 s to choose a stimulus, otherwise the screens turn off and the mouse will have to launch a trial again. In case of a correct response, the mouse has 15 s to retrieve the pellet. Otherwise, the aversive light is turned on for 5 seconds. After completing a trial, the mouse could not launch another one within the next 5 s.*

Supplementary Figure S4. Average number of days required to complete the different phases of the task. *n = 26 per group. Bar: median. Circles: individual data.*

Supplementary references

1. Raftery, A. E. Bayesian Model Selection in Social Research. *Sociol. Methodol.* **25**, 111 (1995).
2. Gruner, P. & Pittenger, C. Cognitive inflexibility in Obsessive-Compulsive Disorder. *Neuroscience* **345**, 243–255 (2017).
3. Dajani, D. R. & Uddin, L. Q. Demystifying cognitive flexibility: Implications for clinical and developmental neuroscience. *Trends Neurosci.* **38**, 571–578 (2015).
4. Bicks, L. K., Koike, H., Akbarian, S. & Morishita, H. Prefrontal Cortex and Social Cognition in Mouse and Man. *Front. Psychol.* **6**, (2015).
5. Uylings, H. B. M., Groenewegen, H. J. & Kolb, B. Do rats have a prefrontal cortex? *Behav. Brain Res.* **146**, 3–17 (2003).
6. Wise, S. P. Forward Frontal Fields: Phylogeny and Fundamental Function. *Trends Neurosci.* **31**, 599–608 (2008).
7. Seamans, J. K., Lapish, C. C. & Durstewitz, D. Comparing the prefrontal cortex of rats and primates: Insights from electrophysiology. *Neurotox. Res.* **14**, 249–262 (2008).
8. Bunge, S. A. & Zelazo, P. D. A Brain-Based Account of the Development of Rule Use in Childhood. *Curr. Dir. Psychol. Sci.* **15**, 118–121 (2006).
9. Ghahremani, D. G., Monterosso, J., Jentsch, J. D., Bilder, R. M. & Poldrack, R. A. Neural Components Underlying Behavioral Flexibility in Human Reversal Learning. *Cereb. Cortex* **20**, 1843–1852 (2009).
10. Hamilton, D. A. & Brigman, J. L. Behavioral flexibility in rats and mice: contributions of distinct frontocortical regions. *Genes Brain Behav.* **14**, 4–21 (2015).
11. Tsuchida, A., Doll, B. B. & Fellows, L. K. Beyond Reversal: A Critical Role for Human Orbitofrontal Cortex in Flexible Learning from Probabilistic Feedback. *J. Neurosci.* **30**, 16868–16875 (2010).
12. Wallis, J. D. Cross-species studies of orbitofrontal cortex and value-based decision-making. *Nat. Neurosci.* **15**, 13–19 (2012).
13. Heilbronner, S. R., Rodriguez-Romaguera, J., Quirk, G. J., Groenewegen, H. J. & Haber, S. N. Circuit-Based Corticostriatal Homologies Between Rat and Primate. *Biol. Psychiatry* **80**, 509–521 (2016).
14. Schoenbaum, G., Setlow, B. & Gallagher, M. Orbitofrontal cortex: modeling prefrontal function in rats. in *The Neuropsychology of Memory* 463–477 (Guilford Press, 2002).
15. Öngür, D. & Price, J. L. The Organization of Networks within the Orbital and Medial Prefrontal Cortex of Rats, Monkeys and Humans. *Cereb. Cortex* **10**, 206–219 (2000).
16. Menzies, L. *et al.* Integrating evidence from neuroimaging and neuropsychological studies of obsessive-compulsive disorder: The orbitofronto-striatal model revisited. *Neurosci. Biobehav. Rev.* **32**, 525–549 (2008).
17. Remijnse, P., Marjan, M., Balkom, A. & et al. REduced orbitofrontal-striatal activity on a reversal learning task in obsessive-compulsive disorder. *Arch. Gen. Psychiatry* **63**, 1225–1236 (2006).
18. Bussey, T. J. *et al.* New translational assays for preclinical modelling of cognition in schizophrenia: The touchscreen testing method for mice and rats. *Neuropharmacology* **62**, 1191–1203 (2012).

19. Brigman, J. L., Graybeal, C. & Holmes, A. Predictably irrational: assaying cognitive inflexibility in mouse models of schizophrenia. *Front. Neurosci.* **4**, (2010).
20. O'Leary, J. D., O'Leary, O. F., Cryan, J. F. & Nolan, Y. M. A low-cost touchscreen operant chamber using a Raspberry Pi™. *Behav. Res. Methods* (2018) doi:10.3758/s13428-018-1030-y.
21. Mishra, J. & Gazzaley, A. Cross-species Approaches to Cognitive Neuroplasticity Research. *NeuroImage* **131**, 4–12 (2016).
22. Horner, A. E. *et al.* The touchscreen operant platform for testing learning and memory in rats and mice. *Nat. Protoc.* **8**, 1961–1984 (2013).
23. Sarter, M. & Berntson, G. G. Tapping artificially into natural talents. *Trends Neurosci.* **22**, 300–301 (1999).
24. Sarter, M. Animal cognition: defining the issues. *Neurosci. Biobehav. Rev.* **28**, 645–650 (2004).
25. Ragland, J. D. *et al.* CNTRICS Final Task Selection: Long-Term Memory. *Schizophr. Bull.* **35**, 197–212 (2009).
26. Sorge, R. E. *et al.* Olfactory exposure to males, including men, causes stress and related analgesia in rodents. *Nat. Methods* **11**, 629–632 (2014).
27. Guarnieri, D. J. *et al.* Gene Profiling Reveals a Role for Stress Hormones in the Molecular and Behavioral Response to Food Restriction. *Biol. Psychiatry* **71**, 358–365 (2012).
28. Neely, C., Lane, C., Torres, J. & Flinn, J. The Effect of Gentle Handling on Depressive-Like Behavior in Adult Male Mice: Considerations for Human and Rodent Interactions in the Laboratory. *Behav. Neurol.* **2018**, (2018).
29. Remmelink, E., Smit, A. B., Verhage, M. & Loos, M. Measuring discrimination- and reversal learning in mouse models within 4 days and without prior food deprivation. *Learn. Mem.* **23**, 660–667 (2016).
30. Torquet, N. *et al.* Social interactions impact on the dopaminergic system and drive individuality. *Nat. Commun.* **9**, 3081 (2018).
31. Maroteaux, G. *et al.* Lack of anticipatory behavior in Gpr88 knockout mice showed by automatized home cage phenotyping. *Genes Brain Behav.* e12473 (2018) doi:10.1111/gbb.12473.
