# Supplementary material for: A cross-species assessment of behavioral flexibility in compulsive disorders
Source: Commun Biol. 2021 Jan 21;4:96. doi: 10.1038/s42003-020-01611-y (PMC7820021; doi:10.1038/s42003-020-01611-y)
Supplement: Supplementary file 2 — Description of Supplementary Files [file 42003_2020_1611_MOESM2_ESM.pdf]

Description of Additional Supplementary Files

File Name: Supplementary Data 1
Description: Humans raw data used to generate Figure 2a

File Name: Supplementary Data 2
Description: Mice raw data used to generate Figure 2a

File Name: Supplementary Data 3
Description: Humans raw data used to generate Figure 2b to Figure 4

File Name: Supplementary Data 4
Description: Mice raw data used to generate Figure 2b to Figure 4
