# Supplementary material for: A cross-species assessment of behavioral flexibility in compulsive disorders
Source: Commun Biol. 2021 Jan 21;4:96. doi: 10.1038/s42003-020-01611-y (PMC7820021; doi:10.1038/s42003-020-01611-y)
Supplement: Supplementary file 3 — Supplementary Data 1 [file 42003_2020_1611_MOESM3_ESM.pdf]

OCD	Trial-10	Trial-9	Trial-8	Trial-7	Trial-6	Trial-5	Trial-4	Trial-3	Trial-2	Trial-1	Trial1	Trial2	Trial3	Trial4	Trial5	Trial6	Trial7	Trial8	Trial9	Trial10
1	0,67	0,73	0,71	0,76	1	1	1	1	1	1	0,05	0,75	0,95	1	0,75	0,68	0,74	0,84	0,71	0,47
1	0,75	0,69	0,71	0,78	1	1	1	1	1	1	0,1	0,5	0,8	0,75	0,8	0,9	0,63	0,56	0,56	0,67
1	0,88	0,76	0,78	0,83	1	1	1	1	1	1	0	0,9	1	0,9	0,85	0,85	0,65	0,76	0,69	0,67
1	0,8	0,8	0,65	0,67	1	1	1	1	1	1	0,05	0,45	0,8	1	0,75	0,85	0,67	0,71	0,8	0,93
1	0,89	0,78	0,67	0,5	1	1	1	1	1	1	0,15	0,75	0,7	0,75	0,55	0,65	0,79	0,74	0,68	0,68
1	0,67	0,8	0,76	0,56	1	1	1	1	1	1	0	0,75	0,8	0,95	0,85	0,75	0,68	0,89	0,88	0,88
1	0,64	0,57	0,6	0,83	1	1	1	1	1	1	0,05	0,55	0,75	0,9	0,85	0,7	0,7	0,56	0,35	0,71
1	0,69	0,56	0,72	0,74	1	1	1	1	1	1	0,05	0,85	0,95	1	0,75	0,7	0,83	0,71	0,82	0,87
1	0,75	0,71	0,88	0,68	1	1	1	1	1	1	0	0,35	0,9	0,85	0,75	0,8	0,89	0,94	0,69	0,8
1	0,79	0,69	0,61	0,61	1	1	1	1	1	1	0,05	0,75	0,95	0,9	0,8	0,95	0,94	0,78	0,63	0,79
1	0,69	0,53	0,47	0,9	1	1	1	1	1	1	0,1	0,55	0,7	0,9	0,9	0,95	0,55	0,53	0,78	0,71
1	0,93	0,88	0,7	0,55	1	1	1	1	1	1	0,1	0,55	0,75	0,95	0,85	0,8	0,9	0,9	0,73	0,6
1	0,75	0,63	0,82	0,71	1	1	1	1	1	1	0,1	0,9	1	1	0,8	0,75	0,59	0,71	0,88	0,73
1	0,67	0,94	0,53	0,84	1	1	1	1	1	1	0	0,7	0,85	0,85	0,8	0,6	0,74	0,68	0,78	0,61
1	0,57	0,71	0,64	0,5	1	1	1	1	1	1	0,05	0,8	0,8	0,85	0,8	0,58	0,81	0,73	0,79	0,5
1	0,78	0,89	0,83	0,7	1	1	1	1	1	1	0,1	0,3	0,7	1	0,9	0,85	0,7	0,79	0,79	0,72
1	0,71	0,57	0,71	0,7	1	1	1	1	1	1	0	0,3	0,6	0,75	0,85	0,7	0,7	0,83	0,67	0,73
1	0,8	0,81	0,71	0,65	1	1	1	1	1	1	0	0,95	1	0,95	0,9	0,8	0,63	0,6	0,79	0,75
1	0,67	0,79	0,74	0,7	1	1	1	1	1	1	0,1	0,45	0,7	0,8	0,8	0,75	0,75	0,6	0,75	0,6
1	0,88	0,56	0,82	0,79	1	1	1	1	1	1	0	0,65	0,85	1	0,9	0,8	0,72	0,75	0,67	0,8
1	0,73	0,81	0,56	0,63	1	1	1	1	1	1	0	0,9	0,75	0,85	0,65	0,95	0,75	0,81	0,87	0,93
1	0,69	0,73	0,67	0,82	1	1	1	1	1	1	0,05	0,35	0,85	0,9	0,85	0,7	0,79	0,71	0,59	0,81
1	0,73	0,88	0,53	0,45	1	1	1	1	1	1	0,1	0,7	0,85	0,9	0,9	0,75	0,7	0,78	0,88	0,69
1	0,57	0,5	0,4	0,61	1	1	1	1	1	1	0,15	0,5	0,65	0,9	0,75	0,9	0,74	0,53	0,86	0,64
1	0,63	0,61	0,63	0,85	1	1	1	1	1	1	0,05	0,65	0,7	0,85	0,75	0,75	0,85	0,7	0,95	0,83
1	0,72	0,61	0,79	0,85	1	1	1	1	1	1	0	0,7	0,75	1	0,95	0,75	0,63	0,68	0,67	0,78
1	0,61	0,89	0,89	0,74	1	1	1	1	1	1	0,3	0,4	0,55	0,7	0,6	0,7	0,7	0,5	0,75	0,55
1	0,69	0,56	0,61	0,7	1	1	1	1	1	1	0,1	0,55	0,8	0,7	0,8	0,65	0,85	0,79	0,63	0,63
1	0,69	0,71	0,65	0,63	1	1	1	1	1	1	0,1	0,35	0,75	0,95	0,7	0,75	0,7	0,84	0,8	0,71
1	0,59	0,88	0,84	0,79	1	1	1	1	1	1	0,05	0,5	0,8	0,95	0,85	0,75	0,71	0,82	0,81	0,63
1	0,78	0,78	0,78	0,61	1	1	1	1	1	1	0	0,65	0,75	0,9	0,8	0,7	0,83	0,76	0,82	0,59
1	0,67	0,47	0,5	0,68	1	1	1	1	1	1	0	0,8	0,9	0,9	0,65	0,8	0,75	0,82	0,63	0,56
1	0,65	0,59	0,65	0,78	1	1	1	1	1	1	0,05	0,85	0,95	1	0,75	0,58	0,65	0,82	0,82	0,71
1	0,77	0,62	0,6	0,79	1	1	1	1	1	1	0	0,75	0,95	1	0,95	0,9	0,79	0,56	0,6	0,73
1	0,84	0,68	0,6	0,65	1	1	1	1	1	1	0,05	0,65	0,95	1	0,8	0,75	0,7	0,65	0,9	0,9
1	0,83	0,5	0,63	0,63	1	1	1	1	1	1	0,3	0,4	0,7	0,9	0,5	0,53	0,58	0,63	0,53	0,74
1	0,68	0,89	0,74	0,75	1	1	1	1	1	1	0,05	0,55	0,9	1	0,75	0,65	0,65	0,74	0,63	0,79
1	0,62	0,47	0,69	0,63	1	1	1	1	1	1	0,05	0,3	0,95	1	0,9	0,95	0,89	0,63	0,86	0,5
1	0,79	0,53	0,56	0,63	1	1	1	1	1	1	0,2	0,5	0,6	0,8	0,85	0,95	0,88	0,75	0,69	0,47
1	0,56	0,5	0,5	0,74	1	1	1	1	1	1	0,1	0,55	0,7	0,9	1	0,8	0,63	0,83	0,75	0,88
0	0,4	0,54	0,67	0,82	1	1	1	1	1	1	0	0,15	0,8	0,9	0,95	0,95	0,95	0,89	0,75	0,67
0	0,76	0,74	0,89	0,8	1	1	1	1	1	1	0	0,15	0,75	0,95	0,85	0,75	0,65	0,55	0,8	0,83
0	0,61	0,56	0,68	0,63	1	1	1	1	1	1	0,2	0,5	0,75	0,85	0,7	0,75	0,8	0,7	0,63	0,84
0	0,5	0,72	0,79	0,75	1	1	1	1	1	1	0	0,65	0,75	1	0,8	0,8	0,9	0,68	0,72	0,76
0	0,93	0,5	0,75	0,78	1	1	1	1	1	1	0,1	0,5	0,65	0,75	0,8	0,85	0,82	0,67	0,64	0,43
0	0,8	0,69	0,76	0,5	1	1	1	1	1	1	0	0,75	0,95	0,95	0,75	0,9	0,79	0,78	0,81	0,8
0	0,93	0,88	0,72	0,83	1	1	1	1	1	1	0,05	0,15	0,8	0,9	0,65	0,75	0,89	1	0,88	0,93
0	0,6	0,76	0,68	0,75	1	1	1	1	1	1	0,05	0,8	0,9	0,95	0,8	0,8	0,65	0,74	0,59	0,73
0	0,79	0,68	0,58	0,5	1	1	1	1	1	1	0,1	0,4	0,55	0,55	0,6	0,85	0,8	0,63	0,63	0,84
0	0,47	0,68	0,6	0,8	1	1	1	1	1	1	0,15	0,75	0,85	0,8	0,55	0,55	0,8	0,9	0,74	0,53
0	0,69	0,75	0,94	0,68	1	1	1	1	1	1	0	0,9	1	0,95	0,85	0,8	0,67	0,75	0,79	0,86
0	0,83	0,67	0,61	0,79	1	1	1	1	1	1	0	0,75	1	1	0,65	0,75	0,68	0,84	0,84	0,95
0	0,69	0,88	0,75	0,63	1	1	1	1	1	1	0,05	0,55	0,75	1	0,85	0,8	0,75	0,73	0,57	0,86
0	0,89	0,67	0,7	0,6	1	1	1	1	1	1	0	0,8	0,95	1	0,75	0,7	0,65	0,83	0,88	0,63
0	0,57	0,6	0,5	0,67	1	1	1	1	1	1	0,15	0,55	0,85	0,75	0,85	0,85	0,89	0,74	0,72	0,75
0	0,71	0,75	0,78	0,7	1	1	1	1	1	1	0,05	0,25	0,9	1	0,9	0,8	0,8	0,65	0,75	0,93
0	0,82	0,72	0,61	0,37	1	1	1	1	1	1	0,15	0,7	0,9	0,75	0,8	0,65	0,7	0,89	0,79	0,72
0	0,85	0,85	0,73	0,72	1	1	1	1	1	1	0	0,6	0,95	1	0,85	0,75	0,72	0,94	0,79	0,71
0	0,6	0,29	0,54	0,71	1	1	1	1	1	1	0,05	0,35	0,85	0,95	0,95	0,95	0,89	1	0,78	0,71
0	0,94	0,56	0,56	0,63	1	1	1	1	1	1	0,1	0,4	0,65	0,9	0,8	0,85	0,74	0,83	0,69	0,69
0	0,63	0,56	0,53	0,79	1	1	1	1	1	1	0,15	0,4	0,6	0,85	0,75	0,9	0,79	0,79	0,88	0,93
0	0,75	0,75	0,81	0,71	1	1	1	1	1	1	0	0,95	0,9	1	0,8	0,8	0,74	0,65	0,76	0,76
0	0,63	0,59	0,89	0,83	1	1	1	1	1	1	0	0,7	0,9	0,85	0,8	0,7	0,79	0,63	0,79	0,74
0	0,6	0,65	0,67	0,63	1	1	1	1	1	1	0,2	0,45	0,7	0,7	0,8	0,7	0,7	0,89	0,83	0,88
0	0,74	0,68	0,6	0,75	1	1	1	1	1	1	0,1	0,55	0,85	0,9	0,7	0,75	0,8	0,68	0,56	0,61
0	0,53	0,74	0,58	0,68	1	1	1	1	1	1	0,05	0,8	0,85	0,95	0,7	0,8	0,68	0,58	0,68	0,74
0	0,73	0,82	0,56	0,85	1	1	1	1	1	1	0	0,6	0,8	1	0,85	0,75	0,9	0,89	0,76	0,8
0	0,88	0,69	0,53	0,76	1	1	1	1	1	1	0	0,45	0,75	0,85	0,8	0,8	0,68	0,63	0,61	0,67
0	0,71	0,79	0,79	0,83	1	1	1	1	1	1	0	0,95	1	1	0,85	0,65	0,61	0,73	0,6	0,73
0	0,82	0,65	0,83	0,63	1	1	1	1	1	1	0,15	0,6	0,9	0,95	0,9	0,7	0,84	0,76	0,75	0,75
0	0,92	0,64	0,67	0,72	1	1	1	1	1	1	0	0,2	0,8	0,9	0,95	0,85	0,8	0,94	0,8	0,86
0	0,81	0,69	0,69	0,67	1	1	1	1	1	1	0	0,55	1	1	0,8	0,7	0,61	0,71	0,76	0,76
0	0,76	0,83	0,68	0,68	1	1	1	1	1	1	0,05	0,3	0,75	0,85	0,85	0,75	0,68	0,79	0,82	0,88
0	0,69	0,85	0,79	0,78	1	1	1	1	1	1	0	0,3	1	1	0,8	0,85	0,79	0,82	0,88	0,94
0	0,63	0,78	0,68	0,74	1	1	1	1	1	1	0	0,45	0,65	0,9	0,65	0,75	0,85	0,75	0,61	0,5
0	0,71	0,76	0,76	0,5	1	1	1	1	1	1	0	0,6	0,45	0,75	0,8	0,8	0,89	0,71	0,65	0,69
0	0,79	0,8	0,67	0,79	1	1	1	1	1	1	0	0,15	0,6	0,95	0,95	1	1	1	1	0,93
0	0,42	0,74	0,63	0,79	1	1	1	1	1	1	0,1	0,1	0,3	0,75	0,65	0,6	0,6	0,65	0,85	0,89
0	0,53	0,65	0,72	0,74	1	1	1	1	1	1	0,05	0,25	0,55	0,85	0,85	0,65	0,7	0,58	0,65	0,76
0	0,75	0,76	0,71	0,71	1	1	1	1	1	1	0	0,75	1	1	0,7	0,85	0,59	0,53	0,71	0,63
