# Supplementary material for: A cross-species assessment of behavioral flexibility in compulsive disorders
Source: Commun Biol. 2021 Jan 21;4:96. doi: 10.1038/s42003-020-01611-y (PMC7820021; doi:10.1038/s42003-020-01611-y)
Supplement: Supplementary file 5 — Supplementary Data 3 [file 42003_2020_1611_MOESM5_ESM.pdf]

TOC	Checkers	MeanErrorRe	MeanRanSC	MeanRanSC1	MeanTrialsTo	MeanTrialsTo	MeanNumPer	OCIRtotal	OCIRlavage	OCIRvérificati	OCIRobsessio	OCIRaccumula	OCIRordre	OCIRneutralis	YBOCS
Healthy	Healthy	2.3	0.83333	3.5714	14.9	15.75	0	6	1	0	1	3	1	0	
Healthy	Healthy	2.15	0.59028	4.1667	14.85	9	0	1	0	0	0	1	0	0	
Healthy	Healthy	1.7	0.61189	6.0714	14.45	10.25	0	14	0	0	2	5	5	2	
Healthy	Healthy	2.05	4.172	14.318	17.2	19.5	0.3	9	0	0	0	3	6	0	
Healthy	Healthy	2.05	3.1292	7.3864	15.45	15.25	0	6	1	0	1	0	4	0	
OCD	"Non-checker	1.9	6.1356	13.244	23.15	18.5	0.095238	39	11	6	11	0	9	2	33
Healthy	Healthy	1.75	3.7942	6.521	19.15	23	0	13	3	2	2	2	4	0	
Healthy	Healthy	2.35	6.8586	10.833	26.55	14.5	0.40909	1	0	0	0	1	0	0	
Healthy	Healthy	1.85	1.7345	1.1364	22.1	14.25	0.375	0	0	0	0	0	0	0	
Healthy	Healthy	1.6	6.0343	3.7351	29.15	27	0.13636	5	1	0	0	2	2	0	
Healthy	Healthy	2.7	4.9268	13.25	23.7	26.5	0.57895	0	0	0	0	0	0	0	
OCD	"Checkers"	1.4	20.333	9.0939	50.45	25.75	0.34579	40	5	10	6	3	10	6	32
OCD	"Non-checker	1.7	8.1207	7.4786	22.5	13.5	0.12	44	12	9	5	6	9	3	29
OCD	"Checkers"	1.6	3.5068	11.251	21.35	26.75	0.26087	24	0	10	5	5	4	0	19
OCD	"Non-checker	1.95	13.17	6.6667	31.55	13.5	0.52778	36	12	4	8	0	12	0	31
Healthy	Healthy	1.7	0	10.833	16.15	8.5		1	0	0	0	1	0	0	
OCD	"Checkers"	1.75	6.4761	15.607	23.65	61.5	0.27027	32	2	8	6	6	8	2	23
OCD	"Non-checker	1.8	3.0428	3.3333	20.55	18.5	0.090909	25	6	0	3	5	7	4	19
Healthy	Healthy	1.75	8.2594	7.1314	29.55	14.25	0.77419	0	0	0	0	0	0	0	
Healthy	Healthy	1.45	7.4549	10.863	23.35	9.5	0.22727	2	0	0	0	2	0	0	
OCD	"Checkers"	1.4	15.646	13.157	30	13.75	0.61702	33	3	7	4	9	10	0	26
OCD	"Checkers"	2.15	7.1177	7.2917	21.7	24	0.1875	38	5	7	8	3	6	9	23
Healthy	Healthy	1.95	4.5049	7.2727	21	18.75	0.15385	3	0	0	0	0	3	0	
Healthy	Healthy	2.15	3.3944	17.146	22.9	17.25	0.125	6	0	0	0	3	3	0	
Healthy	Healthy	1.7	9.0155	5.0195	31.35	26	0.16279	3	2	0	1	0	0	0	
OCD	"Non-checker	1.7	5.2922	7.1767	24.15	16.25	0.11111	13	6	0	3	1	3	0	24
Healthy	Healthy	1.6	4.9253	6.3492	21.3	18.25	0.25	0	0	0	0	0	0	0	
OCD	"Checkers"	1.6	3.9114	8.4182	30.95	18	0.26087	38	12	11	3	0	2	10	36
Healthy	Healthy	1.45	12.212	15.861	34.1	40.25	0.22449	5	0	0	0	2	3	0	
Healthy	Healthy	1.8	5.2485	0	28.6	13	0.28571	1	0	0	0	0	2	0	
Healthy	Healthy	2.3	7.4037	17.06	28.2	15.5	0.31034	0	0	0	0	0	0	0	
Healthy	Healthy	1.25	2.1166	11.563	25.45	26.25	0.63636	10	2	2	0	0	4	2	
OCD	"Checkers"	1.45	2.0849	16.766	20.65	23.75	0	39	10	8	1	7	10	3	29
Healthy	Healthy	1.55	4.9642	8.8993	30.4	30	0.074074	6	0	0	0	1	4	1	
OCD	"Checkers"	1.7	3.461	5.303	23.15	19.25	0	25	0	5	6	5	7	2	25
OCD	"Checkers"	1.45	11.989	12.097	37.4	40.25	0.16667	26	0	12	2	5	1	6	20
OCD	"Checkers"	1.3	16.405	18.305	60.7	21.75	0.36082	21	0	10	3	4	4	0	22
OCD	"Checkers"	1.55	9.9632	8.8095	38.7	22.25	0.19565	33	5	9	8	6	5	0	24
OCD	"Non-checker	1.6	0.9199	4.2572	26.2	20.75	0	38	5	0	10	7	5	11	20
Healthy	Healthy	2	2.6632	7.668	28.15	25	0	0	0	0	0	0	0	0	
OCD	"Non-checker	1.7	4.7095	16.071	20.9	14	0	22	1	1	6	0	9	5	28
OCD	"Non-checker	1.4	4.2759	2.5	25.15	14.75	0.30769	26	11	1	1	6	5	2	33
OCD	"Non-checker	1.55	3.3922	10.268	32.7	19.5	0.33333	44	12	6	11	10	4	1	25
Healthy	Healthy	1.6	4.0366	7.7381	30.4	19.75	0.13333	0	0	0	0	0	0	0	
Healthy	Healthy	1.15	12.539	14.309	48.35	28.5	0.24658	9	0	0	0	2	6	1	
OCD	"Non-checker	1.25	1.4113	8.8889	19.8	20	0.16667	33	9	3	12	0	6	3	20
OCD	"Non-checker	1.9	4.7902	5.9524	31.7	22.25	0.33333	28	12	4	7	0	3	2	22
Healthy	Healthy	1.4	7.0358	9.1919	31.3	21.75	0.14286	0	0	0	0	0	0	0	
Healthy	Healthy	1.45	1.2411	0	22	13.5	0.2	0	0	0	0	0	0	0	
OCD	"Checkers"	1.6	4.4631	8.6193	28.95	40	0.058824	24	7	7	8	1	1	0	25
Healthy	Healthy	1.25	3.3185	4.6998	26.2	19	0.25	16	3	3	2	3	4	1	
OCD	"Checkers"	1.5	3.1682	2	27.15	15.5	0.33333	33	7	10	8	5	3	0	19
OCD	"Checkers"	1.5	1.1908	6.9792	28.65	29.5	0	43	10	10	7	5	7	4	30
OCD	"Non-checker	1.15	1.7133	6.8045	27.65	30.5	0.1	27	12	3	3	0	9	0	33
OCD	"Non-checker	1.3	7.3596	6.164	37.05	25.25	0.096774	33	12	4	8	3	5	1	27
Healthy	Healthy	1.3	9.3024	5.4911	37.75	31	0.51515	3	0	3	0	0	0	0	
Healthy	Healthy	1.4	1.5278	5.3571	21	15	0.5	0	3	0	0	0	0	0	
Healthy	Healthy	1.35	1.4881	2.5	24.95	13.5	0.2	7	4	0	0	3	0	0	
OCD	"Checkers"	1.2	2.3438	3.9474	33.05	17.25	0.15385	17	1	6	4	1	4	1	22
OCD	"Non-checker	1.15	3.5987	6.8643	26.25	26.5	0.13333	10	0	0	7	1	2	0	33
OCD	"Checkers"	1.25	8.7078	8.5492	42.35	37.25	0.36735	34	3	9	10	3	6	3	29
OCD	"Non-checker	1.5	1.2489	6.5768	30.85	21.5	0.125	37	12	1	5	8	10	1	24
OCD	"Non-checker	1.25	1.5774	1.7857	26.1	17	0.2	36	11	0	1	10	10	4	37
Healthy	Healthy	1.9	4.0492	7.8283	25.95	17.75	0.058824	3	0	3	0	0	0	0	
Healthy	Healthy	1.45	3.8266	8.8889	28.9	19.75	0	2	0	0	0	1	1	0	
Healthy	Healthy	1.25	3.5404	5.364	30.6	28.25	0.35714	2	0	0	1	0	1	0	
Healthy	Healthy	1.25	0.48998	5.2778	31.65	18.75	0	5	0	1	0	3	1	0	
OCD	"Checkers"	1.65	1.328	5.3571	31.7	15.75	0	29	2	9	3	2	9	4	21
OCD	"Checkers"	1.35	4.6253	10.887	31.7	42.75	0.052632	28	0	8	3	8	9	0	17
OCD	"Non-checker	1.3	0.33333	4.7619	21.05	19.25	0	14	7	0	2	3	2	0	26
OCD	"Checkers"	1.1	2.7208	10.322	20.9	32.5	0	47	12	12	3	4	12	4	21
OCD	"Checkers"	1	3.3554	1.6667	32.1	30	0.26667	30	9	8	4	4	5	0	20
OCD	"Non-checker	1.45	0.74179	5.3322	24.55	25.25	0	23	10	1	3	1	4	4	19
Healthy	Healthy	1.25	1.8561	6.9444	32.9	20.75	0	0	0	0	0	0	0	0	
Healthy	Healthy	1.1	1.3453	2.3539	24.95	35.5	0	0	0	0	0	0	0	0	
OCD	"Checkers"	1.1	2.9868	6.1395	33.95	33.25	0	21	0	3	5	4	6	3	21
OCD	"Non-checker	1.05	0.40741	4.4408	22.5	16	0	34	4	5	8	3	8	6	23
Healthy	Healthy	1.1	1.4527	0	30.7	14	0	4	0	0	2	0	2	0	
Healthy	Healthy	1.05	1.1033	4.7727	29.15	15	0	4	0	0	0	3	1	0	
Healthy	Healthy	1.25	0.90433	4.7009	28.65	22.25	0	8	1	0	3	3	1	0	
