# Supplementary material for: A cross-species assessment of behavioral flexibility in compulsive disorders
Source: Commun Biol. 2021 Jan 21;4:96. doi: 10.1038/s42003-020-01611-y (PMC7820021; doi:10.1038/s42003-020-01611-y)
Supplement: Supplementary file 6 — Supplementary Data 4 [file 42003_2020_1611_MOESM6_ESM.pdf]

Cluster	Is_KO	Bouts	GroomingPer	P4_toFirstRev	P4_R1RanSC	P4_meanTrial	P4_meanRan	P4_meanCase	P4_meanPerc	P4_TrialsFirst	P4_TrialsLast	P4_TrialsPerHour
WT	WT	14	12.457	569	53.953	588.4	47.184	0.48704	9.7628	692	667	14.568
WT	WT	6	1.785	343	41.667	449	49.322	0.45416	4.4584	351	726	13.005
WT	WT	8	3.7038	159	34.146	315	46.602	0.82436	30.602	532	674	13.136
WT	WT	5	76.154	507	45.775	244.4	43.874	0.52571	36.064	450	634	11.682
WT	WT	12	4.2817	335	62.893	325.8	40.899	0.57291	53.554	706	711	14.129
WT	WT	3	63.842	549	39.227	427.2	41.55	0.68789	10.668	362	887	13.561
WT	WT	8	4.5322	534	68.387	466.2	46.993	0.48951	25.811	702	629	15.917
WT	WT	13	4.4545	219	39.535	372	43.19	0.53767	20.395	544	771	13.678
WT	WT	2	0.28917	381	45.378	379.2	44.024	0.55155	25.976	526	785	14.786
WT	WT	2	0.8675	450	29.31	441.8	45.504	0.43589	16.843	562	733	14.772
WT	WT	5	0.55617	781	44.403	670.2	46.71	0.54587	19.53	412	766	15.136
WT	WT	6	20.949	394	44.355	516.6	36.999	0.55378	38.178	327	675	12.456
WT	WT	3	1.4388	323	36.364	320	47.726	0.39495	29.767	706	679	15.023
WT	WT	4	1.6057	483	47.17	338.8	40.759	0.72479	28.835	389	798	13.522
WT	WT	11	15.46	364	48.322	455.8	43.581	0.60059	19.954	386	732	12.893
WT	WT	2	0.1445	324	38.806	322.8	37.053	0.39994	42.493	409	748	13.366
WT	WT	3	6.6722	457	42.162	229.8	37.49	0.65093	37.929	467	679	12.26
WT	WT	6	25.875	599	55.376	558.8	49.103	0.48508	13.054	405	904	16.551
WT	WT	3	5.5587	503	35.043	430	47.721	0.51775	30.571	647	935	16.791
WT	WT	6	4.1613	452	48.466	854.4	48.638	0.57181	11.476	387	885	16.46
WT	WT	0	0	457	39.13	408.8	42.75	0.69174	28.236	297	717	13.026
WT	WT	3	1.454	371	43.478	450.6	46.351	0.52517	23.29	371	766	13.811
WT	WT	6	13.639	624	46.866	825.2	43.511	0.48575	23.352	420	691	12.873
WT	WT	3	3.6388	1094	49.367	290.8	40.187	0.72481	50.067	235	997	8.3816
WT	WT	0	0	418	50	338.4	46.573	0.68576	6.1084	177	668	11.105
WT	WT	6	32.283	391	54.422	352.4	44.58	0.58529	34.965	416	756	13.128
impaired KO	KO	16	81.854	386	40.113	588	49.312	0.51271	13.347	630	1246	21.739
unimpaired K	KO	7	33.089	260	30.556	854.4	41.799	0.66581	14.949	471	738	15.208
unimpaired K	KO	12	17.863	595	47.451	473	43.809	0.51395	24.551	649	833	16
impaired KO	KO	16	6.1003	332	40.123	703.8	44.713	0.5172	15.929	645	740	17.995
unimpaired K	KO	2	0.26717	160	27.16	395.2	37.435	0.40856	38.862	552	973	15.822
impaired KO	KO	18	47.347	642	46.154	479.6	47.236	0.33813	12.246	567	911	18.204
impaired KO	KO	4	1.3792	1160	51.927	1055.8	47.095	0.45654	13.433	1046	595	13.061
impaired KO	KO	33	30.648	794	45.995	1326	50.837	0.32267	2.9861	837	1265	24.502
impaired KO	KO	9	20.392	441	46.667	809.6	47.506	0.41194	13.557	722	635	16.032
impaired KO	KO	26	15.755	456	46.701	409.6	50.615	0.48237	12.708	882	1149	22.159
unimpaired K	KO	18	16.144	247	44.444	399.2	40.72	0.59242	42.697	918	1130	20.963
impaired KO	KO	19	55.244	268	41.25	574.8	47.293	0.37481	20.71	141	665	12.721
unimpaired K	KO	8	4.3668	304	36.735	287.2	44.566	0.55714	31.025	680	947	17.4
unimpaired K	KO	4	12.496	232	37.838	334	37.765	0.46182	48.001	662	849	16.397
impaired KO	KO	11	5.2167	495	42.021	919.8	46.76	0.39977	9.9922	253	1085	19.15
unimpaired K	KO	15	25.934	144	30	245.8	40.126	0.49941	34.884	687	848	15.782
impaired KO	KO	8	11.713	457	35.955	1001.4	50.152	0.27994	3.3347	363	773	17.513
unimpaired K	KO	14	17.273	317	42.515	325.4	41.678	0.2721	20.673	560	802	14.087
impaired KO	KO	8	43.727	880	40.755	580.4	49.098	0.41478	5.9953	737	654	16.232
unimpaired K	KO	10	61.739	769	44.048	434.8	44.899	0.4659	17.139	937	675	19.362
impaired KO	KO	7	28.698	410	38.389	513.6	46.612	0.37688	8.9592	844	860	18.848
unimpaired K	KO	11	11.439	453	38.06	424.4	38.763	0.44383	35.041	978	869	18.796
unimpaired K	KO	13	35.784	327	42.975	178.8	42.058	0.56978	33.792	185	693	8.0861
unimpaired K	KO	7	28.994	157	44.444	244	39.661	0.38382	40.601	517	681	12.633
unimpaired K	KO	4	1.0112	531	49.289	646.2	43.07	0.47175	27.581	220	704	14.87
unimpaired K	KO	15	35.423	181	34.545	209.2	40.721	0.47407	31.099	365	608	9.5859
