# Supplementary material for: A cross-species assessment of behavioral flexibility in compulsive disorders
Source: Commun Biol. 2021 Jan 21;4:96. doi: 10.1038/s42003-020-01611-y (PMC7820021; doi:10.1038/s42003-020-01611-y)
Supplement: Supplementary file 7 — Reporting Summary [file 42003_2020_1611_MOESM7_ESM.pdf]

nature research

Corresponding author(s): DPBR COMMSBIO-20-0143A

Last updated by author(s): Nov 17, 2020

Reporting Summary

Nature Research wishes to improve the reproducibility of the work that we publish. This form provides structure for consistency and transparency in reporting. For further information on Nature Research policies, see our Editorial Policies and the Editorial Policy Checklist.

Statistics

For all statistical analyses, confirm that the following items are present in the figure legend, table legend, main text, or Methods section.

n/a	Confirmed	
☐	☒	The exact sample size (*n*) for each experimental group/condition, given as a discrete number and unit of measurement
☐	☒	A statement on whether measurements were taken from distinct samples or whether the same sample was measured repeatedly
☐	☒	The statistical test(s) used AND whether they are one- or two-sided<br>*Only common tests should be described solely by name; describe more complex techniques in the Methods section.*
☐	☒	A description of all covariates tested
☐	☒	A description of any assumptions or corrections, such as tests of normality and adjustment for multiple comparisons
☐	☒	A full description of the statistical parameters including central tendency (e.g. means) or other basic estimates (e.g. regression coefficient) AND variation (e.g. standard deviation) or associated estimates of uncertainty (e.g. confidence intervals)
☒	☐	For null hypothesis testing, the test statistic (e.g. *F*, *t*, *r*) with confidence intervals, effect sizes, degrees of freedom and *P* value noted<br>*Give P values as exact values whenever suitable.*
☐	☒	For Bayesian analysis, information on the choice of priors and Markov chain Monte Carlo settings
☒	☐	For hierarchical and complex designs, identification of the appropriate level for tests and full reporting of outcomes
☐	☒	Estimates of effect sizes (e.g. Cohen's *d*, Pearson's *r*), indicating how they were calculated

Our web collection on statistics for biologists contains articles on many of the points above.

Software and code

Policy information about availability of computer code

Data collection	The recorded videos were manually analysed for self-grooming using Kinovea v0.8.15. The human version of the reversal learning task was administered in a computerized version coded in MatLab R2013b (MathWorks) using the Psychophysics Toolbox v3 (http://psychtoolbox.org). The mouse version of the reversal learning task was coded in MED-PC IV (Med Associates).
Data analysis	Bayesian analyses were performed in JASP v0.9.2. We coded in MatLab R2013b (MathWorks) a recursive algorithm based on MatLab functions provided by Gallistel and colleagues to search for putative change points in individual cumulative records of mouse performance. All other analyses were performed in SPSS v25 (IBM).

For manuscripts utilizing custom algorithms or software that are central to the research but not yet described in published literature, software must be made available to editors and reviewers. We strongly encourage code deposition in a community repository (e.g. GitHub). See the Nature Research guidelines for submitting code & software for further information.

Data

Policy information about availability of data

All manuscripts must include a data availability statement. This statement should provide the following information, where applicable:

- Accession codes, unique identifiers, or web links for publicly available datasets
- A list of figures that have associated raw data
- A description of any restrictions on data availability

The data that support the findings of this study are available from the corresponding author upon reasonable request. Source data for main figures 2 to 4 are provided with the publication.

Field-specific reporting

Please select the one below that is the best fit for your research. If you are not sure, read the appropriate sections before making your selection.

☐ Life sciences ☒ Behavioural & social sciences ☐ Ecological, evolutionary & environmental sciences

For a reference copy of the document with all sections, see nature.com/documents/nr-reporting-summary-flat.pdf

Behavioural & social sciences study design

All studies must disclose on these points even when the disclosure is negative.

Study description	Quantitative cross-species behavioural experimental study
Research sample	40 french OCD patients (mean age: 40.15±13.22 years old ; M/F : 15/25), undergoing an SSRI treatment for 28 of them, were recruited through an online advertisement posted on a patient association's website (AFTOC) and among a cohort of severe patients followed in the psychiatric department of Albert Chenevier Hospital. 40 french healthy comparison subjects (mean age: 40.28±13.59 years old ; M/F : 15/25), free of any current psychiatric or neurological disorder and subsequent medications, were recruited through an online advertisement posted on an information web site dedicated to cognitive research (RISC) ; and were matched individually according to age, sex, handedness, school education as well as for IQ.<br>52 C57BL/6J male mice (26 Sapap3-null (KO) and 26 age matched wildtype (WT) littermates), 6-7 months old, were used. The mice were born, weaned (at post-natal day 21) and raised in the animal facility of the Brain and Spine Institute.
Sampling strategy	Due to the use of bayesian statistics, no sample size calculation was performed. Indeed, the notion of power is not relevant in the Bayesian framework. Thus the sample size was determined on the basis of the sample sizes usually included in the literature.
Data collection	Clinical data were collected through both self- and hetero-questionnaires. The human behavioural task was administered by a computer equipped with a 17" TFT monitor and a regular keypad. Only the researcher was present during the task administration. Grooming data were acquired through video recordings and analyzed manually. The mouse version of the behavioural task was administered by a customly modified automatized operant chamber with almost no intervention of the researcher (only 3 minutes per day for weighing).<br>The researcher was not blind to the study hypothesis.
Timing	Human data was collected between January 05, 2015 and April 10, 2017. Mouse data was collected between April 08, 2016 and November 29, 2017.
Data exclusions	No data were excluded from the analyses.
Non-participation	No participants declined participation/dropped out.
Randomization	The participants were not allocated into experimental groups. All participants went through the same experimental procedure.

Reporting for specific materials, systems and methods

We require information from authors about some types of materials, experimental systems and methods used in many studies. Here, indicate whether each material, system or method listed is relevant to your study. If you are not sure if a list item applies to your research, read the appropriate section before selecting a response.

Materials & experimental systems

n/a	Involved in the study	
☒	☐	Antibodies
☒	☐	Eukaryotic cell lines
☒	☐	Palaeontology and archaeology
☐	☒	Animals and other organisms
☐	☒	Human research participants
☒	☐	Clinical data
☒	☐	Dual use research of concern

Methods

n/a	Involved in the study	
☒	☐	ChIP-seq
☒	☐	Flow cytometry
☒	☐	MRI-based neuroimaging

Animals and other organisms

Policy information about studies involving animals; ARRIVE guidelines recommended for reporting animal research

Laboratory animals	Fifty-two C57BL/6J male mice (26 Sapap3-null (KO) and 26 age matched wildtype (WT) littermates), 6-7 months old, were used.
Wild animals	The study did not involve wild animals.

April 2020

Field-collected samples	The study did not involve samples collected from the field.
Ethics oversight	Each animal experiment was approved by the Ethics committee Darwin/N°05 (Ministère de l'Enseignement Supérieur et de la Recherche, France) and conducted in agreement with institutional guidelines, in compliance with national and European laws and policies (Project n° 00659.01).

Note that full information on the approval of the study protocol must also be provided in the manuscript.

Human research participants

Policy information about studies involving human research participants

Population characteristics	See above.
Recruitment	OCD patients were recruited through an online advertisement posted on a patient association's website (AFTOC) and among a cohort of severe patients followed in the psychiatric department of Albert Chenevier Hospital. Healthy comparison subjects were recruited through an online advertisement posted on an information web site dedicated to cognitive research (RISC).
Ethics oversight	The protocol for human participants was approved by the Medical Ethical Review Committee of the Pitié-Salpêtrière Hospital (ID RCB n° 2012-A01460-43). All the participants gave their informed consent prior to the beginning of the study.

Note that full information on the approval of the study protocol must also be provided in the manuscript.
