# Supplementary material for: A cross-species assessment of behavioral flexibility in compulsive disorders
Source: Commun Biol. 2021 Jan 21;4:96. doi: 10.1038/s42003-020-01611-y (PMC7820021; doi:10.1038/s42003-020-01611-y)
Supplement: Supplementary file 4 — Supplementary Data 2 [file 42003_2020_1611_MOESM4_ESM.pdf]

KO	Trial-100	Trial-99	Trial-98	Trial-97	Trial-96	Trial-95	Trial-94	Trial-93	Trial-92	Trial-91	Trial-90	Trial-89	Trial-88	Trial-87	Trial-86	Trial-85	Trial-84	Trial-83	Trial-82	Trial-81	Trial-80	Trial-79	Trial-78	Trial-77	Trial-76	Trial-75	Trial-74	Trial-73	Trial-72	Trial-71	Trial-70	Trial-69	Trial-68	Trial-67	Trial-66	Trial-65	Trial-64	Trial-63	Trial-62	Trial-61	Trial-60	Trial-59	Trial-58	Trial-57	Trial-56	Trial-55	Trial-54	Trial-53	Trial-52	Trial-51	Trial-50	Trial-49	Trial-48	Trial-47	Trial-46	Trial-45	Trial-44	Trial-43	Trial-42	Trial-41	Trial-40	Trial-39	Trial-38	Trial-37	Trial-36	Trial-35	Trial-34	Trial-33	Trial-32	Trial-31	Trial-30	Trial-29	Trial-28	Trial-27	Trial-26
1	0,6	1	1	0,2	0,8	0,8	0,4	0,8	1	0,8	0,2	0,4	0,8	0,6	1	0,4	0,6	0,8	0,4	0	0,6	0,8	0,8	0,4	0,6	0,4	0,8	0,6	0,8	0,4	0,8	1	0	0,6	0,6	0,4	0,6	0,8	0,8	0,8	0,6	0,8	0,8	0,4	0,8	0,8	0,6	0,4	0,6	0,2	1	0,6	0,8	0,8	0,6	0,8	1	0	0,8	0	1	0,8	0,6	0,6	1	1	0,6	0,2	0,8	0,4	0,8	0,8	0,6	0,6	1
1	0,6	0,8	0,6	0,6	0,6	0,4	0,6	1	1	0,8	0,8	0,4	0,8	1	0,6	0,8	0,8	0,4	0,8	0,4	0,6	0,8	0,4	0,4	0,6	1	0,4	0,8	0,8	0,8	0,8	0,8	0,6	0,6	0,8	0,8	0,8	0,6	0,8	0,6	0,4	0,8	1	0,6	0,8	0,6	0,4	0,6	1	0,6	0,2	0,6	0,4	0,6	1	1	0	0,6	0,8	0	0,8	0,6	0,8	0,6	0,8	0,8	1	0,6	0,8	1	0,6	0,6	0,8	0,8	0,6
1	0,6	0,6	0,4	0,8	0,4	0,6	0,4	0,6	0,8	0,4	0,6	0,6	0,8	0,8	0,8	0,6	0,4	0,8	0,6	1	0,6	0,6	0,6	0,6	0,6	0,6	0,4	0,4	1	0,8	0,8	0,8	0,6	0,6	1	0,4	0,6	0,6	0,8	0,2	0,2	0,2	0,4	0,6	0,4	0,6	0,6	0,4	0,6	0,4	0,4	0,8	0,6	0,8	0,4	0,4	0,8	0,2	0,6	0	1	1	0,8	1	0,4	1	0,6	1	0,6	1	0,6	0,6	0,6	0,8	0,8
1	0,6	0,8	0,4	0,6	0,6	0,2	1	0,4	0,6	0,6	0,6	0,6	0,8	0,6	0,8	0,6	0,8	0,6	0,8	1	0,6	0,4	0,6	0,6	0,6	0,8	0,8	0,8	0,4	0,6	1	0,6	0,4	0,4	1	0,6	0,2	0,8	0,4	0,4	0,8	0,6	0,6	1	0,4	0,6	0,6	1	0,8	0,4	0,6	0,6	0,6	0,6	0,8	0,2	0,4	1	0,8	0	0,6	0,8	1	0,4	0,6	0,6	0,6	0,8	0,8	1	0,6	0,8	0,6	0,8	0,8
1	0,6	0,6	0,8	0,4	0,6	0,4	0,8	0,6	0,4	0,8	0,8	0,6	0,6	0,8	0,6	0,6	0,4	0,6	0,8	0,8	0,6	0	0,8	0,6	0,8	0,8	0,4	0,4	0,6	0,4	0,2	0,6	0,6	0,2	0,6	0,8	1	0,6	0,4	0,8	0,6	0,6	0,6	0,8	0,4	1	0,8	1	0,6	0,8	0,6	0,4	0,6	0,6	0,8	0,6	0,4	0,8	0,4	0	0,8	0,6	0,6	0,8	0,8	0,6	0,8	1	0,6	0,4	0,6	0,6	0,8	0,8	0,6
1	0,4	0,4	0,6	0,4	0,6	0,8	0,6	1	0,4	1	0,2	0,8	0,6	1	0,8	0,8	0,6	0,8	0,2	0,6	0,6	1	0,8	0,6	0,8	0,8	0,8	0,8	0,6	0,4	0,8	0,8	0,4	0,6	0,8	1	0,6	1	0,4	0,6	0,6	0,4	0,6	0,4	0,8	0,4	0,8	0,6	1	0,4	0,8	0,6	0,6	0,4	0,8	1	1	0,6	0,6	0	0,6	0,8	0,8	0,4	0,6	1	0,6	1	0,6	0,8	0,6	0,6	0,8	0,8	0,8
1	0,4	0,4	1	0,6	0,8	0,8	0,8	0,4	1	0,8	0,6	0,6	1	0,6	1	0,8	1	0,8	0,4	0,8	0,4	1	0,8	0,8	0,2	1	0,6	0,4	1	0,8	0,4	0,6	0,6	1	0,6	0,8	0,6	0,6	0,6	1	0,2	0,8	0,4	0,4	0,8	1	1	0,2	0,4	0,8	0,8	0,8	0,6	0,6	0,6	0,4	0,6	1	0,8	0	1	0,6	1	1	0,6	0,8	0,6	0,8	1	0,6	0,4	1	0,6	1	0,6
1	1	0,6	1	0,8	0,4	0,8	0	0,8	0,8	0,6	0,6	0,8	1	0,4	0,8	0,6	0,6	1	0,8	0,6	0,6	0,8	1	0,6	0,8	0,6	0,8	0,4	0,8	0,8	0,2	0,8	0,8	1	0,4	1	1	0,4	0,8	0,6	0,8	0,4	0,4	0,8	0,4	0,4	0,8	0,4	0,4	0,4	0,6	0,4	0,6	0,8	0,8	0,4	1	0,2	0,6	0	1	0,8	0,8	0,4	0,8	0,6	1	0,6	0,6	1	1	0,8	0,4	0,8	0,8
1	0,6	1	0,6	0,2	0,6	0,8	0,6	0,6	0,6	0,6	0,4	1	0,8	0,4	1	0,8	0,6	0,6	0,4	0,6	0,4	0,8	0,6	1	0,6	0,6	1	0,2	0,6	0,4	0,8	0,8	0,8	0,6	1	0,8	0,4	0,8	0,6	0,8	0,8	0,8	0,4	0,4	1	0,8	0,8	0,4	0,4	1	0,8	0,6	0,6	0,4	0,8	0,2	0,6	0,8	0,4	0	0,8	0,8	0,6	1	1	0,8	0,8	0,8	0,6	1	0,8	0,6	0,6	1	1
1	0,6	0,6	0,2	0,8	0,4	1	1	1	0,8	0,6	0,8	0,8	0,8	0,6	0,8	0,6	0	0,8	0,6	0,6	0,6	0,4	0,4	0,6	0,6	0,6	0,8	0,8	0,6	0,8	0,4	0,8	0,8	0,6	0,2	0,8	0	0,6	0,4	0,4	0,8	0,6	0,6	0,6	0,8	0,6	0,6	1	0,6	0,8	0,8	0,6	0,6	0,8	0,4	0,6	0,4	0,6	0,8	0	1	0,8	0,6	1	0,6	1	0,8	0,6	0,4	0,6	1	0,6	0,6	0,4	0,8
1	0,6	0,4	0,4	0,8	0,2	0,6	0,8	0,4	0,6	0,8	0,8	0,6	0,4	0,6	0,6	0	0,6	0,8	0,2	0,6	0,6	0,6	0,4	0,8	0,8	1	0,4	1	0,6	1	0,4	0,8	0,4	0,2	0,6	0,8	1	0,4	0,6	0,4	0,8	1	0,6	0,8	0,6	0,8	0,4	0,8	1	0,6	1	0,8	0,4	1	0,2	0,6	0,8	0,8	0,2	0	0,8	1	0,8	0,8	0,8	0,6	0,6	0,8	0,6	0,6	0,8	0,8	1	0,6	0,6
1	1	0,6	0,6	0,6	0,6	0,6	0,6	0,4	0,4	0,8	0,6	0,4	0,2	0,8	0,4	0,6	0,8	0,6	0,4	0,6	0,8	0,6	0,6	0,8	0,4	0,6	0,8	0,6	0,6	0,4	0,8	0,8	0,8	0,2	1	0,8	0,4	0,2	0,6	0,6	0,8	0,6	0,4	0,4	0,8	0,8	0,6	0,2	0,2	0,2	1	0,4	0,4	0	0,6	0,6	0,6	0,4	0,8	0	1	0,8	0,8	0,6	0,6	0,8	0,8	0,8	0,8	0,8	0,6	0,6	1	0,8	0,6
1	0,6	0,2	0,2	0,2	0,4	0,4	0,6	0,4	0,2	0,8	0,4	0,6	0,6	0,6	0,6	0,6	0,6	0,2	0,6	0,4	0,8	0,6	0,4	0,8	1	0,2	0,4	0,8	0,6	0	0,8	0,8	0,6	0,8	0,6	0,6	1	0,6	1	0,2	0,6	0,8	0,6	0,8	0,8	0,4	0,4	0,8	0,6	0	0,4	0,6	0,8	0,6	0,4	0,6	0,4	0,6	0,4	0	1	0,6	1	0,4	0,6	0,6	0,8	0,8	0,4	0,6	0,6	1	0,2	0,6	0,8
1	0,2	0,4	0,6	0,6	0,8	0,6	0,6	0,4	0,6	0,8	0,6	0,6	1	0,4	0,4	0,8	0,6	0,6	1	0,4	1	0,8	0,6	0,8	0,6	0,4	0,6	0,6	1	0,8	0,4	0,4	0,4	0,8	0,4	0,6	1	0,8	0,4	0,8	0,4	0,6	0,4	0,8	0,4	1	1	0,6	0,6	1	0,8	1	0,4	0,6	0,6	0,4	0,8	0,4	0,8	0	0,8	0,8	0,6	0,4	1	0,6	0,8	1	0,6	0,4	0,6	0,8	0,6	1	0,8
1	0,2	0,6	0,6	0,6	0,2	0,8	0,6	0,6	0,6	0,6	0,2	0,4	0,6	0,6	0,8	0,8	0,6	0,6	0,6	0,2	0,8	0,4	1	0,4	0,6	0,8	0,8	0,6	0,6	0,2	0,8	0,4	0,6	0,2	0,6	0,6	0,6	0,8	0,6	0,2	0,4	0,6	0,8	0,6	0,8	0,4	0,6	0,8	0,8	0,6	1	0,4	1	0	1	1	0,6	0,8	0,2	0	1	0,8	0,6	0,8	1	0,8	0,4	1	0,8	0,6	0,8	0,8	0,6	0,8	0,8
1	0,8	0,4	0,4	0,4	0,8	0,2	0,6	0,8	0,8	0,6	0,6	0,6	0,6	0,4	0,6	0,8	1	0,2	1	1	0,4	0,6	0,6	0,8	0,6	1	0,4	0,4	0,4	0,6	0,4	0,6	0,6	0,4	0,6	0,6	0,6	0,4	0,8	0,6	0,8	0,2	0,8	0,6	0,4	0,8	1	0,6	0,6	0,6	1	0,4	0,6	0,6	0,4	1	1	0,6	0,8	0	1	0,4	0,6	1	0,6	0,8	0,8	0,8	1	0,4	1	1	0,8	1	0,8
1	0,8	0,6	0,4	1	0,8	0,6	0,6	0,4	0,6	0,8	0,4	0,6	0,6	1	0,4	1	0,8	0,8	0,8	0,8	0,8	0,8	0,8	0,4	0,6	0,6	0,4	0,6	1	0,8	0,4	0,6	0,4	0,8	0,6	0,6	0,8	0,2	1	0,6	0,6	1	0,8	0,8	0,4	0,4	1	0,6	0,6	0,4	0,8	0,4	0,8	0,4	0,8	0,6	0,8	0	1	0	1	0,8	0,6	1	0,2	0,8	0,6	0,8	1	0,8	0,6	0,8	0,8	0,8	0,8
1	0,6	0,8	1	0,6	1	0,6	0,4	0,8	0,8	0,6	0,4	0,6	0,4	1	0,6	0,6	0,6	1	0,4	0,6	0,4	0,6	0,4	0,6	0,8	0,6	0,4	0,8	0,6	0,6	0,6	0,8	0,4	0,6	0,6	0,8	0,4	0,4	0,8	0,6	0,4	0,8	0,4	0,8	0,6	1	0,6	1	0,4	0,8	0,8	0,4	1	0,8	0,4	0,6	0,8	0,6	0,8	0	0,6	0,6	0,6	1	0,6	1	0,6	0,4	0,8	1	1	1	0,4	1	0,8
1	0,4	0,8	0,2	1	0,6	0,8	0,8	0,6	0,8	0,6	0,4	0,8	0,4	0,8	0,6	0,6	0,8	0,2	0,8	0,8	0,6	0,4	0,2	0,6	0,6	0,6	0,4	0,6	0,6	0,8	1	0,6	0,6	0,6	0,6	0,2	0,8	0,6	0,8	0,4	0,8	0,4	0,6	0,6	0,2	0,8	0,4	0,6	0,8	0,6	0,6	0,4	0,6	0,6	0,6	0,8	0,8	0,6	0,4	0	1	1	0,8	0,6	0,6	1	0,8	0,6	0,6	0,8	0,8	0,8	1	1	1
1	0,8	0,6	0,2	0,4	0,6	0,4	0,6	0,6	1	0,6	0,4	0,8	0,6	0,6	0,8	0,8	1	0,6	0,4	0,8	1	0,6	0,8	0,8	0,8	0,6	0,8	0,4	0,6	0,8	0,6	0,6	0,4	0,6	0,8	1	0,8	0,4	0,6	0,6	0,4	0,8	0,8	0,8	0,4	0,8	0,4	0,4	0,6	0,6	0,8	0,6	1	0,8	0,2	0,4	0,8	0,8	0,6	0	1	0,6	0,6	0,6	0,6	0,8	1	0,8	1	1	1	0,8	0,8	0,8	0,8
1	1	0,8	1	0,6	0,4	1	0,2	0,4	0,6	0,6	0,8	0,2	0,4	0,8	0,2	0,6	0,6	0,8	1	1	0,6	1	0,6	0,8	0,6	0,6	1	0,6	0,4	0,8	0,8	0,6	0,8	0,8	0,6	0,8	0,6	0,4	0,6	0,6	0,8	0,8	0,8	0,6	0,8	0,8	0,6	0,6	0,6	0,8	0,6	0,8	0,8	0,6	0,8	0,4	0,6	0,6	1	0	1	0,2	0,8	1	1	0,8	0,6	0,6	1	1	0,2	0,8	1	0,8	0,6
1	0,8	0,6	1	0,6	0,6	0,8	0,8	0,2	0,8	0,6	0,8	0,4	0,4	0,8	0,8	0,4	0,8	0,6	0,6	0,4	1	0,8	0,8	1	0,8	0,4	0,6	1	0,4	0,4	0,4	0,8	0,6	0,8	0,6	0,6	0,6	0,8	0,8	0,4	1	0,6	0,8	0,4	0,6	1	0,8	0,6	0,6	0,6	0,8	0,8	0,6	0,8	0,6	0,8	0,6	0,6	0,8	0	1	0,4	0,6	0,6	0,8	0,4	1	1	0,8	0,4	0,6	0,8	0,6	1	1
1	0,6	0,2	0,4	0,6	0,4	0,8	0,8	0,4	0,6	0,2	0,6	0,6	0,6	0,6	0,4	0,4	0,6	0,6	0,6	0,6	0	0,6	0,4	0,8	0,4	0,2	0,8	0,2	0,8	0,8	0,6	0,8	0,6	0,8	0,8	0,6	0,6	0,8	0,2	0,8	0,6	0,6	0,8	0,6	1	0,8	0,4	0,6	0,4	0,2	0,8	0,8	0,8	0,6	0,8	0,2	0,8	1	0,2	0	1	0,8	1	1	0,4	1	0,6	0,8	0,8	0,8	0,6	0,8	0,6	0,8	0,2
1	0,8	0,6	0,2	0,2	0,4	0,2	0,2	0,4	0,6	0,2	0,6	0,2	0,8	1	0,4	0,2	0,8	0	0,8	0,2	0,6	0,6	1	0,6	0,4	0,6	0,6	0,4	0,8	0,4	0,8	0,6	0,8	0,4	0,6	0,6	0,4	0,6	1	0,6	0,6	0,4	0,6	0,8	0,8	0,6	0,4	0,6	0,8	0,4	1	0,4	0,8	0,4	0,6	0,4	0,6	0,2	0,8	0	0,4	0,6	0,4	1	0,6	0,8	0,6	1	0,6	0,6	0,6	0,6	1	0,6	1
1	0,8	1	0,4	0,6	0,2	1	0,6	0,8	0,4	0,8	0,6	0,8	1	0,6	0,8	0,8	0,8	0,6	0,8	0,4	0,6	1	0,8	0,8	0,6	0,6	0,8	0,4	0,6	0,6	0,6	1	0,6	0,4	0,8	0,6	1	0,6	0,6	0,4	0,2	0,6	0,4	0,6	0,8	0,4	0,6	1	0,4	0,8	0,8	0,4	0,8	0,6	0,6	0,8	0,6	1	0,6	0	0,8	0,8	0,6	0,8	0,6	1	0,8	1	1	0,8	0,8	0,6	0,8	0,6	0,8
1	0	0,6	0,6	0,4	0,4	0,4	0,4	0	0,6	0,8	0,4	0,4	0,8	0,4	0,4	0,2	0,8	0,6	0,8	0,4	0	1	0,6	0,6	0,4	0,2	0,6	0,6	0,8	0,8	0,8	0,8	0,2	0,8	0,8	0,6	0,6	0,2	0,8	0,6	0,6	0,4	0,8	0,6	0,6	0,4	0,8	0,8	0,4	0,8	0,8	0,6	0,8	0,6	0,6	0,8	0,8	0,4	0,8	0	0,4	0,6	0,8	0,6	0,8	1	0,4	1	0,4	0,6	0,8	1	1	1	0,8
0	0,8	0,8	0,6	0,4	0,8	0,4	0,6	0,8	0,8	0,8	0,4	0,6	0,2	1	0	1	0,8	0,6	0,8	0,2	0,8	0,6	0,8	0,8	0,6	0,8	0,4	0,6	1	0,4	0,8	0,8	0,6	0,6	0,6	0,8	0,4	0,8	1	0,4	0,8	0,4	0,6	0,8	0,6	0,6	0,8	0,8	0,4	0,8	0,4	0,6	1	0,6	0,6	0,6	0,6	0,4	1	0	0,6	0,8	0,4	0,8	0,8	0,8	0,4	0,6	0,6	0,8	0,6	0,8	0,6	0,8	0,8
0	0,6	0,8	1	0,6	0,8	1	0,8	0,4	0,4	0,8	0,2	0,8	0,6	0,6	0,2	0,6	0,6	0,8	0,8	0,4	0,8	0,6	0,8	0,6	0,2	0,8	1	0,8	0,8	1	0,2	0,4	0,8	0,6	0,6	0,8	0,2	0,6	0,4	0,6	0,6	0,8	0,4	0,8	0,4	0,8	0,8	0,6	0,8	0,4	0,6	0,6	0,8	1	0,6	0,6	0,6	0,4	0,8	0	0,8	0,8	0,8	1	0,8	0,8	1	0,6	0,8	0,8	1	0,4	0,6	0,8	0,6
0	0,8	0,4	0,2	0,6	0,4	0,8	0,4	0,8	0,4	0,4	0,2	0,2	0,2	0,2	0,2	1	0,6	0,2	0,4	0,8	0,6	0,4	0,4	0,4	0,6	0,6	0,6	0,4	0,4	0,8	0,2	0	0,4	0,6	0,8	0,6	0,8	0,6	0,6	0,4	0,8	0,8	0,4	0,4	0,4	0,6	0,6	0,4	0,6	0,6	0,4	0,6	0,2	0,2	0,4	0,6	0,8	0,8	0,2	0	0,4	0,8	0,4	1	0,8	0,6	0,6	0,8	0,8	1	0,8	1	1	0,6	0,6
0	0,8	0,6	0,6	0,6	0,6	0,4	0,4	0,6	0,6	0,6	0,6	0,6	0,6	0,4	0,6	0,6	0,6	0,2	0,6	0,6	0,4	0,6	0,4	0,6	0,6	0,6	0,6	0,2	0,6	0,4	0,8	0,6	0,2	0,6	0,8	0,6	0,4	0,8	0,4	0,8	0,4	0,4	0,6	0,2	1	1	0,6	0,8	0,6	0,2	0,4	0,8	0,6	1	0,6	0,8	0,8	0,6	0,4	0	0,6	1	0,6	0,8	0,8	0,6	1	0,8	1	0,6	0,6	1	0,6	0,6	0,8
0	0,4	0,6	0,8	0,6	0,6	0,4	0,4	0,8	0,6	1	0,8	0,8	1	0,8	0,8	0,6	0,8	0,8	0,8	0,8	0,4	0,8	0,8	0,8	0,8	0,6	0,8	0,8	0,2	0,8	0,2	0,6	0,6	1	0,8	0,8	0,6	0,6	0,6	0,8	0,6	0,6	0,6	1	1	0,4	0,6	0,8	0,6	0,4	0,6	0,8	0,6	0,8	0,6	0,8	0,6	0,6	0,8	0	0,6	0,8	0,8	0,8	0,2	1	0,6	0,8	0,8	0,8	0,6	0,8	0,4	1	1
0	0,6	0,6	0,6	0,4	1	0,4	0,4	0,6	0,6	0,4	0,6	0,8	0,4	0,6	0,6	0,6	0,8	0,2	0,4	0,6	0,6	0,4	0,6	0,8	0,4	0,6	0,2	0,8	0,8	0,6	0,6	0,8	0,6	1	0,4	0,4	0	1	0,2	0,6	0,8	0,8	0,4	0,4	0,8	0,4	0,8	0,6	0,4	1	0,6	0,2	0,8	1	0,4	0,6	0,6	0,2	1	0	0,8	0,8	1	0,6	0,4	0,8	1	0,6	0,8	0,6	0,4	0,8	0,8	0,8	0,6
0	0,4	0,8	0,6	0,6	0,6	0,6	0,6	0,8	0,4	0,8	0,6	0,4	0,4	0,6	0,8	0,4	1	0,6	0,4	0,8	0,8	0,6	0,8	0,6	0,6	0,8	0,4	0,8	1	0,8	0,2	1	0,6	0,4	1	0,6	1	0,6	1	1	0,4	0,8	0,4	0,6	0,2	0,8	0,6	0,2	1	1	0,6	0,6	1	0,4	0,8	0,4	0,2	0,8	1	0	1	0,8	0,8	0,8	0,6	0,4	1	0,8	0,6	0,4	1	1	0,2	0,8	1
0	1	0,2	1	0,2	1	0,4	1	0,2	0,6	0,6	1	0,6	0,6	0,2	1	0,4	0,6	0,8	0,8	0,6	0,6	0,4	0,6	0,6	0,2	0,6	0,2	0,4	0,4	0,6	0,8	0,4	0,6	0,6	0,4	0,8	0,4	0,6	0,8	0,2	0,4	0,6	1	0,2	0,8	0,6	0,6	0,2	0,8	0,6	1	0,4	0,6	0,4	0,2	0,4	0	1	0,4	0	0,8	1	0,8	0,6	0,2	1	1	0,8	0,8	0,8	0,8	0,6	0,8	0,8	0,8
0	0,6	0,6	0,4	0,6	0,8	1	0,4	0,6	0,8	0,6	0,8	1	0,6	1	0,4	0,6	0,4	0,8	0,8	0,8	0,4	0,4	0,6	0,4	0,4	0,6	0,8	0,4	0,8	0,6	0,4	0,6	0,6	0,6	0,8	0,4	0,4	1	0,8	1	0,8	1	0	0,6	0,8	0,4	0,8	0,6	0,2	0,8	0,8	0,8	0,8	0,6	0,2	0,4	0,2	1	0,8	0	0,8	0,8	0,8	1	0,6	0,6	0,8	1	1	0,8	1	1	0,8	0,8	0,4
0	0,4	0,8	0,6	0,6	0,4	0,6	0,6	0,6	0,6	0,6	0,8	0,4	0,4	0,6	0,8	0,4	0,4	0,6	0,8	0,6	0,4	0,6	0,4	0,6	0,2	0,8	0,6	0,8	0,6	0,8	0,4	0,8	0,4	1	0,6	0,4	0,2	0,8	0,4	0,6	0,8	1	0,6	0,4	0,8	0,6	0,2	0,4	0,8	0,8	0,6	0,4	0,2	0,4	1	0,6	0,2	0,6	1	0	0,8	0,8	0,8	0,8	0,6	0,8	0,8	1	0,8	0,8	0,6	0,8	1	0,6	0,8
0	0,6	0,6	0,4	0,6	0,6	0,8	0,8	0,4	0,6	0,8	0,6	0,8	0,6	0,4	0,8	0,6	0,6	0,8	0,4	0,6	0,6	1	0,4	0,6	1	0,4	0,6	1	0,6	0,6	0,4	1	1	0,8	0,6	0,8	0,6	0,8	0,6	0,4	0,8	0,6	0,4	0,8	1	0,6	1	0,6	0,6	0,6	0,6	0,4	0,8	0,6	0,8	0,6	0,8	0,6	0,2	0	0,8	1	0,4	1	0,8	0,4	1	0,8	0,8	0,6	0,8	0,8	1	0,6	0,6
0	0,6	0,6	0,4	0,8	0,8	0,2	0,4	0,2	0,8	0,8	0,4	0,6	0,4	0,6	0,4	0,8	0,6	0,2	0,8	1	0,8	0,6	0,6	0,6	0,4	0,8	0,4	0,8	0,8	0,8	0,4	0,4	0,8	1	0,6	0,6	0,8	0,8	0,8	0,8	0,8	0,6	0,2	0,6	0,8	0,4	1	0,2	0,4	1	0,8	0,8	0,8	0,6	0,6	0,8	0,6	0,8	0,6	0	0,4	0,6	0,8	0,6	0,8	1	1	0,6	1	0,4	0,8	1	0,8	0,8	0,6
0	0,4	1	0,8	0,6	0,8	0,6	0,4	0,2	0,6	0,6	0,6	0,6	0,2	0,2	0,4	0,4	0,4	0,4	0,8	0,6	0,2	0,4	0,4	0,6	0,8	0,2	0,6	0,4	0,4	0,6	0,6	0,4	0,6	0,4	0,4	0,8	0	0,4	0,2	0,6	0,8	0,2	0,8	0,6	0,6	1	0,4	1	0,6	0,6	0,6	0,6	0,4	0,6	0,8	0,4	0,6	0,6	0,4	0	0,4	0,6	0,8	0,8	0,8	0,6	0,8	0,8	0,6	0,8	0,6	0,8	0,8	0,8	1
0	0,4	0,8	0,4	0,4	0,8	0,6	0,6	0,6	0,6	0,6	0,8	0,6	1	0,6	0,6	0,8	0,6	0,8	0,6	0,6	0,4	0,6	0,8	0,8	0,2	0,2	0,4	0,4	0,4	0,6	0,4	0,8	0,4	0,8	0,4	0,8	0,6	0,8	1	0,6	0,8	0,6	0	0,8	0,4	0,8	0,6	0,6	0,6	0,6	0,4	0,2	0,6	0,6	0,4	0,8	0,6	0,4	1	0	1	0,6	0,8	0,6	0,8	0,8	0,8	0,8	0,8	0,6	0,6	0,4	0,6	0,8	1
0	0,6	0,8	0,6	0,6	0,4	0,8	0,6	0,2	0,6	0,6	0	0,6	0,8	0,8	0,4	0,4	0,6	0,4	0,4	0,8	1	0,4	0,4	0,6	0,6	0,4	0,6	0,4	0,4	0,4	0,6	0,8	0,4	0,6	0,6	0,6	0,4	0,6	0,8	0,8	0,6	0	0	0,4	0,4	0,8	0,4	0,8	0,6	0,6	0	0,6	0,4	0,4	0,8	0,6	0,4	0,8	0,4	0	1	0,4	0,8	1	0,8	0,6	0,8	0,8	0,8	1	0,6	0,8	0,8	0,8	1
0	0,8	0,6	0,6	0,6	0,2	0,4	0,2	0,4	0,4	0,4	0,6	0,6	0,8	0,6	0,6	0,6	0,4	0,4	0,2	0,6	0,6	0,6	0,4	0,8	1	0,8	0,4	0,8	0,8	0,8	1	0,6	0,8	0,6	0,8	1	0,8	0,4	0,4	0,6	1	0,2	0,6	0,8	0,8	0,8	0,6	1	0,4	0,6	1	0,4	0,4	0,4	0,8	1	0,8	0,8	0,6	0	0,8	0,8	0,6	0,8	0,8	0,8	0,8	0,8	0,8	0,8	0,8	0,2	0,8	0,4	0,6
0	0,8	0,8	0,8	0,4	0,4	0,6	0,8	1	0,4	0,8	0,8	0,6	0,6	0,4	0,2	0,4	1	0,4	0,2	0,4	0,8	1	0,6	0,8	0,6	0,4	0,2	0,8	0,8	0,6	0,6	0,4	0,8	0,8	0,8	0,4	0,6	0,6	0,8	0,4	0,2	0,2	0,6	0,4	0,2	1	0,6	0,2	0,4	0,6	0,4	0,6	0,6	0,8	0,4	0,2	0,8	0,8	0,4	0	1	0,8	0,6	0,6	0,8	1	0,8	1	0,8	0,4	0,8	0,8	0,4	1	0,8
0	0,4	0,4	0,4	0,8	0,2	0,6	0,4	0,8	0,6	0,4	1	0,6	0,4	0,8	0,6	0,8	0,6	0	0,8	0,8	0,4	0,6	0,6	0,8	0,8	0,4	0,8	0,4	0,8	0,8	0,4	0,8	0	0,6	0,8	1	0,6	0,8	0,8	0,6	1	0,4	0,8	0,8	0,4	1	0,8	0,8	0,2	0,8	0,6	0,8	0,4	0,4	0,6	0,4	0,8	0,8	1	0	1	0,8	1	0,8	0,6	1	0,8	0,6	0,8	0,8	0,4	0,4	1	0,4	0,8
0	0,2	1	0,6	0,8	1	0,6	0,8	0,8	0,6	0,4	0,4	0,8	0,4	0,6	0,6	0,4	0,6	0,6	0,8	0,8	0,6	0,6	0,4	0,8	0,6	0,2	0,4	1	0,6	0,6	0,8	0,6	0,8	1	0,4	1	0,6	0,6	0,8	0,8	1	0,4	0,6	0,8	0,4	0,4	0,4	0,4	0,8	0,8	0,2	0,6	0,6	0,4	0,6	0,8	0,4	0,8	0,6	0	1	1	0,8	0,6	0,6	0,6	0,8	1	0,8	0,8	1	1	0,6	1	0,2
0	0,8	0,8	0,6	0,6	0,6	0,6	0,4	0,6	0,4	0,4	0,8	0,6	0,8	0,4	0,8	0,6	0,8	0,4	0,6	0,8	0,8	0,6	0,8	0,6	1	0,8	0,4	0,8	0,6	0,6	0,2	0,4	0,8	0,8	0,2	0,4	0,6	0,8	0,8	0,4	0,4	0,4	1	0,4	0,6	0,4	0,8	1	0,8	0,6	0,6	0,6	1	0,2	0,6	0,6	0,8	0,2	1	0	0,4	0,6	1	0,6	0,4	0,6	0,8	1	0,8	0,8	0,6	0,6	0,6	0,8	0,6
0	0,6	0,6	0,8	0,6	0,6	0,4	0,6	0,6	0,4	0,2	0,6	0,8	0,2	0,6	0,4	0,4	0,4	0,4	0,4	0,8	0,4	0,6	0,6	0,8	0,6	0,8	0,6	0,8	0,6	0,4	0,8	1	0,4	0,6	0,6	0,4	0,8	0,6	0,6	1	0,6	0,8	0,6	0,6	0,2	1	0,6	0,6	0,2	0,6	0,4	0,6	0,6	0,8	0,4	0,4	0,6	0,4	0,4	0	0,8	0,4	0,6	0,8	0,8	0,8	1	1	0,6	1	0,4	0,6	0,6	0,6	0,8
0	0,4	0,6	0,2	0,8	0,8	0,8	0,8	0,8	0,4	0,6	0,6	0,8	0,4	0,4	0,8	0,8	0,8	0,4	0,4	0,4	0,2	0,6	0,8	0,6	0,2	1	0,8	0,4	0,4	0,4	0,2	0,8	0,6	0,4	0,8	0,6	0,6	0,4	0,8	0,6	0,6	0,6	0,8	0,8	0,6	0,4	0,8	0,2	0,4	1	0,6	0,4	1	0,8	0,8	1	0,4	0,4	0,8	0	1	0,6	1	1	0,4	0,6	0,8	0,6	1	0,6	0,8	0,8	0,8	0,4	1
0	0,6	1	0,6	0,6	0,4	0,8	0,6	1	0,8	0,8	0,4	0,6	0,6	0,6	0,8	0,6	0,4	0,6	0,4	0	0,6	0,8	1	0,2	0,6	0,8	0,8	0,8	0,4	0,6	0,4	0,4	0,6	0,6	0,8	0,8	0,6	0,2	0,8	0,4	0,6	0,8	0,6	0,8	0,8	0,4	0,8	0,2	0,4	0,8	0,4	0,6	0,6	1	0,6	0,8	0,4	0,8	0,8	0	0,6	0,8	0,6	0,4	1	0,6	0,8	0,8	0,6	0,8	0,8	0,8	0,6	0,8	0,8
0	0,2	1	0,8	0,8	0,2	0,6	0,8	0,8	0,4	0,6	0,4	0,4	0,6	0,6	0,6	0,8	0,8	0,6	0,6	1	0,6	0,4	0,6	0,8	1	0,8	0,8	0,2	0,8	0,6	0,6	0,2	0,8	0,8	0,2	0,2	0,6	0,6	0,6	0,8	0,6	1	0,4	0,8	0,8	0,2	0,8	0,8	0,6	0,6	0,4	0,8	0,2	0,4	0,4	0,8	0,2	0,8	0,2	0	0,6	0,8	0,8	0,4	0,8	0,6	0,6	1	1	1	1	0,8	1	0,6	0,6
0	0	0,8	0,4	1	0,2	0,2	0,4	0,6	0,8	0,6	0,8	0,6	0,6	0,6	0,6	0,2	0,6	0,6	0,6	0,2	0,6	0,6	0,6	0,2	0,4	0,8	0,8	0,4	0,6	0,8	0,6	0,4	1	0,4	0,8	0,4	0,8	0,8	0,8	0,4	0,8	0,4	0,8	0,8	0,4	0,4	0,4	0,6	0,6	0,4	0,8	0,6	0,6	0,8	0,8	1	0,8	0,4	0,4	0	0,8	0,6	1	0,6	0,2	0,6	1	0,6	0,8	0,8	1	0,8	0,8	1	0,8
0	0,6	0,8	0,6	0,6	0,8	0,4	0,4	1	0,6	0,4	0,6	0,6	0,6	0,8	0,2	0,4	0,2	0,2	0,4	0,6	0,6	0,4	0,4	0,4	0,6	0,6	0,6	0,8	0,4	0,4	0,6	0,6	0,6	0,2	0,4	0,6	0,4	0,4	0,4	0,4	0,6	0,8	0,6	0,6	0,6	0,6	0,4	0,6	0,6	0,4	0,8	0,4	0,2	0,2	0,2	0,2	0,6	0,4	0,4	0	0,8	0,8	0,6	0,8	0,6	1	0,8	0,8	0,6	1	0,6	0,2	1	1	0,8

Trial-25	Trial-24	Trial-23	Trial-22	Trial-21	Trial-20	Trial-19	Trial-18	Trial-17	Trial-16
0,8	0,8	0,8	0,8	0,8	1	0,8	1	0,6	0,8
0,2	1	1	1	1	0,8	0,6	1	0,6	1
0,8	1	0,8	1	0,4	0,8	0,8	0,4	0,8	1
0,8	0,8	0,6	0,8	0,8	1	0,8	1	0,8	1
0,8	1	0,8	1	1	0,8	0,8	0,8	1	1
1	0,8	1	0,8	1	0,8	0,8	0,8	0,8	0,8
0,6	0,8	1	0,8	1	0,6	0,6	0,8	0,6	1
0,8	0,4	1	0,6	0,8	1	1	1	0,8	0,4
0,6	0,8	0,4	0,8	0,8	0,8	0,8	0,6	1	0,8
0,8	1	0,8	0,4	0,8	0,8	0,8	0,6	0,8	1
0,8	0,6	1	1	0,8	1	1	0,6	0,6	0,6
0,8	0,8	1	1	1	0,8	0,8	1	1	0,8
0,6	0,8	0,8	0,8	0,8	0,8	1	0,8	0,8	0,8
1	0,6	0,8	1	1	0,4	0,6	0,8	0,8	0,6
0,8	0,6	0,8	1	0,6	0,6	0,8	0,8	1	1
0,6	0,8	0,8	0,8	0,6	0,8	0,8	0,6	0,8	1
0,4	0,8	0,6	0,8	1	1	1	0,4	0,6	0,6
0,6	0,8	0,8	0,8	0,6	0,4	1	0,6	1	1
0,6	0,8	0,2	0,8	1	0,6	0,8	1	0,6	0,8
0,8	0,4	1	0,8	0,8	0,6	0,8	0,6	0,6	0,4
1	0,6	0,8	0,8	1	0,8	0,8	1	0,4	0,6
0,8	1	0,8	0,6	0,8	0,8	1	0,4	1	0,8
0,8	0,8	0,6	1	0,8	1	0,8	0,8	0,8	1
1	0,8	0,6	0,8	0,8	1	0,8	1	0,8	0,8
1	0,4	0,6	0,8	0,8	0,8	1	0,6	1	0,6
0,6	0,8	1	0,8	0,8	1	1	1	1	0,8
0,8	0,8	0,4	1	0,8	0,8	1	1	1	0,8
0,6	1	0,4	0,8	0,4	1	0,8	0,6	1	1
0,4	0,8	0,6	0,4	1	1	1	0,8	1	1
1	0,6	1	0,8	0,4	0,8	0,8	0,8	1	0,8
0,6	0,4	1	0,8	1	0,8	1	0,8	0,8	0,8
1	0,8	1	1	0,6	0,8	0,6	1	0,8	1
0,4	0,6	1	0,4	1	1	1	1	1	0,8
0,2	1	0,4	0,8	0,6	1	1	0,6	0,6	1
0,6	0,8	1	0,8	0,4	0,8	0,8	0,6	0,2	0,4
0,6	0,6	0,6	1	1	1	0,6	0,8	0,8	0,8
1	0,8	0,2	1	0,6	0,4	1	0,8	1	0,8
0,6	1	0,6	0,8	1	0,6	0,6	0,8	1	0,4
0,6	0,8	0,8	1	0,6	0,8	0,8	1	1	0,4
1	1	1	0,8	0,8	0,8	0,6	0,8	1	0,4
1	0,8	0,6	0,6	0,4	0,8	0,8	0,6	0,8	0,6
0,8	0,6	0,8	0,8	0,8	0,8	1	1	0,8	0,8
1	0,6	1	0,8	0,6	0,6	0,8	0,8	1	0,4
0,8	0,6	1	0,4	0,6	1	0,4	1	1	0,8
0,8	0,8	0,6	1	0,4	0,8	0,8	0,6	0,8	0,8
0,8	0,8	1	0,6	1	0,8	1	0,8	0,8	1
0,8	1	1	0,8	1	0,8	1	1	0,8	0,8
0,6	1	1	0,8	0,8	0,6	0,8	0,4	1	0,8
0,8	0,8	1	0,8	0,8	0,8	0,8	1	0,6	0,8
0,4	0,2	1	1	0,8	0,6	1	0,6	0,8	0,4
0,6	0,8	1	0,8	1	0,6	0,8	0,8	1	0,8
1	0,2	1	0,6	0,6	1	0,8	0,6	0,8	1

Trial-15	Trial-14	Trial-13	Trial-12	Trial-11	Trial-10	Trial-9	Trial-8
0,8	0,6	1	1	1	0,8	0,6	1
1	0,4	0,8	0,6	0,8	0,8	1	1
1	0,8	1	0,6	1	0,6	0,8	0,2
0,8	0,8	0,8	0,8	1	1	0,8	0,8
0,4	1	0,8	0,6	0,8	1	1	0,6
0,8	0,8	0,8	0,8	0,6	1	0,6	0,8
0,6	0,8	1	0,8	1	0,6	0,8	1
0,4	1	0,8	0,8	1	0,8	0,8	0,8
0,6	1	0,6	0,6	0,8	0,8	1	0,6
0,4	0,8	1	1	1	1	1	1
0,8	1	1	1	1	0,8	0,4	0,6
1	0,8	1	0,6	0,8	0,6	1	0,6
0,6	1	0,8	1	1	0,8	1	1
1	1	0,8	0,8	1	1	0,8	0,8
0,6	0,8	0,4	0,8	0,8	1	0,8	1
0,6	0,6	1	0,8	0,4	0,8	0,8	1
1	0,8	1	0,6	0,8	1	0,8	0,8
0,8	1	0,8	1	0,6	1	0,8	0,4
0,6	0,8	1	1	0,8	0,2	1	1
0,6	1	0,8	1	0,8	0,6	1	0,6
0,8	0,8	0,8	0,8	1	0,8	0,8	1
0,6	0,4	1	1	1	0,8	0,6	1
0,6	0,8	0,8	0,6	1	0,8	0,6	1
0,8	0,6	1	0,8	1	1	0,8	0,8
0,6	0,8	0,8	1	0,8	0,8	0,4	0,8
0,8	0,8	0,6	1	0,4	1	0,8	0,8
1	0,6	1	0,8	0,8	1	1	0,8
0,8	0,8	0,6	0,8	0,8	0,8	0,8	1
1	0,8	1	1	0,6	0,8	1	1
0,8	0,4	1	0,8	0,6	0,6	1	1
0,8	1	1	0,2	1	1	0,8	1
0,8	1	0,6	0,8	1	1	0,8	0,6
1	0,4	0,8	0,8	1	0,8	0,8	0,4
1	1	0,8	1	0,8	1	0,8	1
0,8	1	1	0,8	0,8	0,8	1	0,8
0,4	0,8	0,8	1	1	0,8	0,8	0,8
0,6	0,8	0,8	0,6	0,8	0,8	1	0,8
0,8	1	0,8	0,8	0,8	1	1	1
1	1	0,6	0,8	0,8	0,8	1	1
0,8	1	0,8	0,8	0,8	0,6	0,6	1
0,8	1	0,8	0,8	1	0,8	0,8	1
1	1	0,8	1	0,6	0,8	1	0,6
0,8	0,8	0,8	0,6	0,8	0,6	0,8	1
1	0,8	0,8	0,6	0,6	1	0,6	1
0,8	0,8	1	0,6	0,4	1	0,8	1
0,6	0,8	0,8	1	0,6	0,8	1	1
1	0,8	0,8	0,4	0,8	0,8	0,6	0,8
0,6	0,6	1	0,8	0,6	1	1	1
1	0,8	0,8	1	0,4	1	0,8	0,8
0,6	1	1	1	0,8	1	1	0,8
0,6	0,6	0,8	1	0,8	0,8	0,8	0,6
0,8	1	0,8	0,8	1	0,8	0,8	1

Trial-7	Trial-6	Trial-5	Trial-4	Trial-3	Trial-2	Trial-1	Trial1	Trial2
0,4	1	1	0,8	1	1	1	0	0
0,8	0,8	0,8	1	0,8	1	1	0,2	0,8
0,8	1	1	0,8	1	0,8	1	0,6	0,4
0,6	1	0,8	0,8	1	0,8	1	0,2	0,4
1	1	0,8	0,8	1	0,8	1	0,2	0,4
0,8	1	0,8	1	0,8	1	1	0,2	0,2
0,8	0,6	1	0,8	0,8	1	1	0	0,8
1	1	1	0,6	0,8	1	1	0,2	0,4
1	1	0,8	0,6	1	1	1	0,2	0,4
1	0,8	0,8	0,8	1	0,8	1	0,2	0,8
0,8	0,6	1	1	0,8	1	1	0	0,2
0,8	0,2	0,6	0,6	1	1	1	0,2	0,2
1	1	1	0,8	1	1	1	0	0,6
0,8	1	1	0,8	0,8	1	1	0,4	0
0,8	1	0,6	1	0,8	1	1	0,2	0,6
0,6	0,8	1	1	1	1	1	0,2	0,6
0,8	1	1	1	0,8	1	1	0,2	0,4
1	0,8	1	0,8	1	1	1	0	0,4
1	0,8	0,8	0,6	0,8	1	1	0,4	0,2
1	0,6	1	1	1	1	1	0	0,6
0,8	0,8	1	0,6	0,8	1	1	0,6	0,2
0,8	1	0,8	1	1	1	1	0	0,2
0,8	0,8	1	1	0,6	1	1	0,2	0,6
0,8	1	1	1	0,8	0,8	1	0,2	0
1	0,6	1	1	1	1	1	0,2	0,6
0,8	0,6	0,6	1	1	0,8	1	0,8	0
1	0,6	0,8	1	1	1	1	0	0,2
1	1	1	0,4	1	1	1	0,2	0,6
0,6	1	0,8	0,8	0,8	0,6	1	0	0
0,6	0,8	1	1	1	1	1	0	0,4
0,6	0,6	1	1	1	1	1	0,2	0,4
0,8	0,6	0,8	0,8	1	1	1	0	0,6
0,8	0,8	1	1	1	1	1	0,2	0
1	0,8	0,4	0,8	1	0,8	1	0	0,4
0,8	0,8	1	1	0,8	1	1	0,2	0,2
0,6	0,6	0,8	1	1	1	1	0,4	0,4
1	1	0,8	1	1	1	1	0	0,4
0,6	1	0,8	1	1	0,8	1	0,6	0
0,8	1	0,6	1	1	0,8	1	0,2	0,2
0,8	0,8	0,8	1	1	1	1	0,4	0,6
1	0,8	1	0,4	1	0,8	1	0,6	0,4
0,6	0,8	1	1	1	1	1	0,2	0,6
1	1	0,8	1	0,8	1	1	0	0,4
1	0,8	1	1	1	1	1	0,2	0,4
1	0,8	1	0,8	1	0,8	1	0,2	0,4
0,8	1	1	1	0,8	1	1	0	0,6
0,6	1	1	0,8	1	0,8	1	0	0,2
1	1	1	0,6	0,6	1	1	0,4	0,2
1	1	0,8	1	0,6	1	1	0	0,2
1	0,6	1	0,8	1	1	1	0,4	0,4
1	1	0,6	1	1	0,8	1	0,2	0,2
0,8	1	0,8	1	0,4	1	1	0,2	0,2

Trial3	Trial4	Trial5	Trial6	Trial7	Trial8	Trial9	Trial10
0,4	0,6	0	0,4	0	0,2	0,2	0,6
0	0,4	0,6	0,4	0,4	0,6	0	0,4
0,6	0,2	0,4	0,2	0,6	0,6	0,2	0,4
0,4	0,2	0,4	0,4	0,2	0,2	0,2	0,6
0	0,2	0,4	0	0,4	0,2	0	0,2
0,6	0,2	0,6	0,2	0,4	0,6	0,4	0,4
0,2	0,2	0,2	0,4	0,4	0,2	0,4	0,2
0,2	0,4	0,2	0,4	0,6	0	0,6	0,8
0,4	0,4	0,4	0,6	0,2	0,4	0,4	0,4
0,2	0,4	0,2	0,4	0,4	0,2	0,4	0,4
0,2	0	0	0,2	0,4	0,4	0,2	0,6
0,6	0	0,8	0,4	0,4	0,2	0,4	0,4
0,4	0,2	0,2	0,6	0,2	0,8	0,2	0,6
0,2	0,4	0	0,8	0,2	0,4	0	0,4
0,6	0,2	0,4	0,8	0,4	0,4	0,4	0,4
0,4	0,6	0	0	0,6	0,4	0,4	0,2
0,4	0,4	0,6	0,6	0,2	0,6	0,4	0,6
0,2	0,4	0,2	0,2	0,6	0,2	0,4	0,2
0,2	0,2	0,6	0,2	0,2	0,4	0,6	0,4
0,4	0,6	0,4	0,2	0,8	0,4	0,8	0,6
0,6	0,8	0,4	0,6	0,2	0,6	0,4	0,4
0,6	0,6	0,4	0,4	0,4	0,4	0,2	0,2
0,2	0,2	0,4	0,2	0,4	0,6	0,2	0,2
0,6	0,4	0,2	0,2	0,2	0,6	0,2	0,2
0	0,2	0,6	0,2	0,8	0,2	0,4	0,4
0,6	0,4	0,4	0,2	0,2	0	0	0,4
0,2	0,8	0,4	0,4	0,6	0	0,6	0,4
0,4	0,4	0,2	0,8	0,4	0,6	0,2	0,6
0,8	0,2	0,2	0,2	0,4	0,4	0,4	0,6
0,4	0,2	0,6	0,2	0,2	0	0,4	0
0	0,2	0,8	0,6	0,4	0	0,4	0,2
0,6	0,2	0,2	0,2	0,2	0,2	0,4	0,4
0,2	0,2	0,2	0	0,6	0	0,2	0
0,2	0,6	0,2	0,2	0,6	0,2	0,2	0,2
0,2	0,6	0	0,8	0,4	0,2	0	0,2
0,6	0,6	0,4	0,4	0,4	0,2	0,4	0,2
0,2	0,4	0,2	0,2	0,4	0,2	0,2	0,6
0,2	0,2	0,2	0	0	0	0,6	0,6
0,4	0,8	0,2	0,4	0	0,4	0	0,4
0,4	0,6	0,2	0,2	0,4	0,6	0	0,6
0	0,4	0,2	0,4	0,4	0,4	0	0,6
0,6	0,2	0	0	0,2	0,6	0	0,2
0,2	0,2	0	0,4	0	0,4	0,2	0,6
0,4	0,4	0,4	0,4	0,4	0,6	0,2	0,4
0,2	0,4	0,4	0,2	0,2	0,2	0,4	0,2
0,4	0,6	0,4	0	0,2	0,6	0	0,2
0,8	0,4	0,6	0,2	0,4	0,4	0	0,2
0,6	0,2	0,6	0,6	0,4	0,4	0,4	0,4
0,4	0,4	0,6	0,2	0,4	0,4	0	0,8
0,4	0,6	0,4	0,6	0,2	0	0,2	0,2
0,2	0,4	1	0,6	0,4	0,8	0,2	0,2
0,4	0,4	0,2	0	0,6	0,2	0,6	0,4

Trial11	Trial12	Trial13	Trial14	Trial15	Trial16	Trial17	Trial18
0	0,4	0,6	0,6	0,2	0,2	0,2	0,4
0,4	0,2	0,6	0,4	0,2	0,4	0,2	0,4
0,4	0,4	0,4	0,2	0,2	0,2	0,2	0,4
0,2	0,6	0,2	0,2	0,4	0	0	0,2
0,4	0,2	0,6	0,2	0,4	0,4	0,4	0,4
0	0,4	0,6	0,6	0,2	0,2	0,2	0,6
0,4	0,4	0,2	0,2	0,6	0	0,4	0,4
0,6	0,6	0,4	0,6	0,6	0,6	0,4	0,6
0	0,4	0,4	0,2	0,8	0,6	0,4	0,4
0,4	0,2	0,2	0,2	0	0,4	0,6	0,2
0,2	0	0	0,4	0,2	0,4	0,4	0
0,8	0,2	0,2	0,6	0	0,4	0,2	0,6
0	0,6	0	0,2	0,4	0,6	0,2	0,2
0,2	0,2	0	0,6	0,4	0,6	0,6	0,4
0	0,4	0,8	0,2	0,6	0,6	0,4	0
0,6	0	0,4	0,6	0,6	0,4	0,2	0,4
0,2	0,2	0,4	0,6	0,2	0,6	0,4	0,2
0,2	0,4	0,4	0,4	0,8	0	0,4	0,8
0,2	0,4	0,2	0,2	0,4	0,6	0,4	0,2
0,2	0	0	1	0,2	0,2	0,6	0,2
0	0,4	0,4	0,4	0,2	0,6	0,2	0,4
0,2	0,2	0,4	0	0,2	0,6	0,2	0,6
0,4	0,2	0,8	0,4	0,4	0,4	0,2	1
0,4	0,2	0,6	0,2	0,4	0	0,2	0,2
0,6	0,4	0,4	0,4	0,6	0,6	0	0,2
0,2	0,4	0,6	0,4	0	0,6	0,2	0,2
0,2	0,6	0,4	0,4	0,6	0,8	0,2	0
0,4	0,4	0,2	0	0,8	0,4	0,8	0,4
0,2	0,6	0,4	0,6	0	0,4	0,4	0,2
0	0,4	0,4	0,6	0,2	0,4	0	0,6
0,4	0	0,2	0	0	0,4	0,2	0,6
0,4	0,2	0,6	0,2	0,8	0,6	0,6	0,2
0,4	0,2	0,6	0,6	0,2	0,6	0,6	0,4
0,6	0,4	0,4	0,4	0,6	0,4	0,2	0
0,8	0	0,2	0,6	0,6	0	0,4	0,2
0,4	0,8	0	0,2	0,8	0,4	0,6	0,4
0,4	0,2	0	0	0,8	0,2	0,2	0,4
0	0,4	0	0,4	0,6	0,4	0,4	0,2
0,8	0,8	0	0,2	0,8	0,2	0	0,6
0,4	0,6	0	1	0,4	0,2	0	0,4
0,2	0,2	0	0,4	0,4	0,4	0,2	0,2
0,2	0,4	0,4	0,4	0,4	0,4	0,8	0,4
0	0,2	0,6	0	0,4	0	0,2	0,8
0,8	0,2	0	0,4	0,2	0,2	0,6	0,6
0,6	0,4	0,4	0,2	0,2	0,8	0,6	0,2
0,2	0,4	0,2	0,4	0	0,4	0,8	0,4
0,2	0,6	0,4	0,4	0,2	0,4	0,2	0,6
0,2	0,6	0,6	0,6	0,6	0,2	0,2	0,8
0,4	0,2	0,2	0,4	0,6	0,2	0,8	0,6
0,2	0,4	0,2	0,2	0,2	0,4	0	0,8
0,4	0,6	0,4	0,6	0,4	0,6	0,6	0,4
0,2	0,6	0,6	0,2	0,2	0,2	0,4	0,6

Trial19	Trial20	Trial21	Trial22	Trial23	Trial24	Trial25	Trial26	Trial27
0,4	0,2	0,8	0,6	0,4	0,6	0,4	0,6	0,6
0,2	0,6	0,2	0,4	0,4	0,2	0,6	0,4	0,4
0,6	0,2	0,2	0,2	0,2	0,6	0,4	0,4	0
0,6	0,2	0,8	0,2	0,4	0,2	0,6	0	0,2
0,4	0,4	0,6	0,6	0,6	0,2	0,6	0,2	0,2
0,2	0,6	0,8	0,4	0,6	0,4	0,4	0,2	0,4
0,4	0,4	0,4	0,2	0,4	0,4	0,4	0,6	0,6
0,2	0,6	0,6	0,2	0,2	0	0,8	0,2	0,4
0	0,6	0,4	0,4	0,6	0	0,8	0,4	0,4
0,6	0,4	0,2	0,6	0,6	0,2	0,6	0,2	0,4
0,2	0,2	0,2	0,2	0,2	0,2	0,2	0,4	0,4
0,6	0,6	0,2	0,8	0,2	0,4	0,4	0,4	0,6
0	0,2	0,4	0,4	0,4	0,4	0,2	0,4	0,2
0,2	0,4	0,6	0,6	0	0,4	0,2	0,4	0,8
0,6	0,2	0,6	0,6	0,4	0,2	0,4	0,4	0,4
0,4	0,4	0,2	0,4	0,6	0,6	0,6	0,6	0,4
0,4	0	0,6	0,6	0,6	0,4	0,8	0,2	0,4
0,6	0,6	0,6	0,2	0,6	0,2	0,2	0,2	0,6
0,6	0,4	0,4	0,2	0,6	0,4	0,6	0,6	0,4
0,6	0,4	0,4	0,6	0,4	0,6	0,4	0,4	0,2
0,4	0,4	0,2	0,6	0,8	0,4	0,2	0,4	0,4
0,4	0,4	0,6	0,8	0,8	0,4	0,2	0	0,6
0,2	0,4	0,6	0,4	0	0,8	0,8	0,2	0,2
0,6	0,4	0,4	0,8	0,4	0,4	0,4	0,6	0,2
0,4	0,2	0,6	0,6	0	0,6	0	0,2	0,4
0,2	0,2	0,4	0,2	0,2	0,4	0,4	0,4	0,2
0,4	0,6	0,6	0	0,6	0	0,8	0,4	0,4
0,2	0,6	0,2	1	0,8	0,4	0,6	0,4	0,4
0,2	0,4	0,2	0,2	0,4	0,4	0,4	0,6	0,2
0,2	0,4	0,2	0	0,8	0,2	0,2	0,2	0,2
0	0,2	0,4	0,4	0,6	0,2	0,4	0,2	0,2
0,4	0,6	0,6	0,4	0,4	0,6	0,6	0,6	0,6
0,2	0,6	0,2	0,4	0,4	0,2	0,4	0,6	0,6
0,8	0	0,4	0,4	0,4	0,4	0,2	0,6	0,6
0,2	0,6	0,2	0	0,6	0,2	0,4	0,4	0,4
0,8	0,2	0,6	0	0,4	0,4	0,4	0,8	0,2
0,8	0,4	0	0,2	0,4	0,6	0,2	0,2	0,4
0,4	0,4	0,4	0,2	0,2	0,8	0,4	0,4	0
0,4	0,4	0,8	0,4	0,8	0,6	0,4	0,8	0,4
0,2	0,4	0,8	0,2	0,2	0,6	0,2	0	0,8
0,4	0	0,6	0,2	0,2	0,6	0,2	0,2	0,2
0,2	0,2	0,4	0,2	0,6	0,2	0,2	0,4	0,2
0	0,4	0,4	0,2	0,6	0	0,6	0,4	0,6
0,4	0,4	0,4	0,2	0,4	0,4	0,6	0,6	0
0,4	0,2	0,6	0,4	0	0,2	0,4	0,4	0,4
0,4	0,2	0,6	0,6	0,2	0,6	0	0,4	0,6
0,6	0,4	0,4	0,6	0,4	0,2	0,8	0,2	0,2
0,2	0,6	0,4	0,2	0,4	0,4	0,4	0,8	0,2
0	0,2	0,6	0,6	0,4	0,2	0	0,8	0,2
0,4	0,6	0,4	0,2	0,2	0,4	0,2	0,2	0,2
0,8	0,8	0,6	0,6	0,6	0,4	0,6	0,4	0,8
0,4	0,2	0,4	0,8	0,4	0,2	0,4	0,2	0,4

Trial28	Trial29	Trial30	Trial31	Trial32	Trial33	Trial34	Trial35
0,6	0,4	0,4	0,6	0,2	0,8	0,6	0,2
0,4	0,6	0,2	0,6	0,6	0,8	0,4	0,2
0,6	0,4	0,2	0,4	0,2	0,4	0,6	0
0,2	0,6	0,6	0,4	0,6	0,6	0,2	1
0,4	0,4	0,2	0,4	0,6	0,6	0,4	0,4
0,6	0,4	0,2	0,8	0,6	0,4	0,6	0,8
0,2	0,2	0,2	0,4	0,4	0,8	0,4	0,6
0,2	0,8	0,4	0,6	0,4	0,8	0,6	0,4
0,6	0,8	0,6	0,4	0,4	0,4	0,6	0,6
0,4	0,4	0,8	0,6	0,4	0,2	0,8	0,2
0,2	0	0,2	0,4	0,4	0,6	0,2	0,8
0,4	0,2	0,4	0,4	0	0,2	0,4	0,8
0,6	0,6	0,2	0,2	0,4	0,6	0,6	0,2
0	0,6	0,2	0,8	0,8	0,6	0,2	0,4
0,6	0,2	0,2	0,4	0,6	0,4	0,2	0,4
0,2	0,8	0,4	0,2	0,6	0,4	0,6	0,6
0,6	0,6	0,8	0,4	0,2	0,6	0,6	0,6
0,8	0,6	0,2	0,2	0,8	0,4	0,6	0,6
0,6	0,4	0,8	0,2	0,6	0,4	0,4	0,4
0,2	0,4	0,4	0,4	0,2	0,8	0,4	0,2
0,6	0,4	0,4	0,8	0,2	0,8	0,8	0,2
0,2	0,6	0,4	0,2	0,6	0,6	0,2	0,8
0,6	0,4	0,6	0,6	0,4	0,6	0,6	0,4
0,6	0,2	0,6	0,4	0,4	0,2	0,2	0,4
0,2	0,4	0,4	0,4	0,4	0,2	0,4	0,2
0	0,2	0,6	0,4	0,6	0,4	0,4	0,2
0,6	0,4	0,2	0,4	0,6	0,2	0,6	0,2
0,4	0,6	0,2	0,4	0,8	0,6	0,6	0,2
0,4	0,4	0,4	0,2	0,8	0,2	0,2	0,6
0,4	0,6	0,6	0,2	0,6	0,4	0,6	0,2
0,4	0,6	0	0,2	0,4	0,4	0	0,4
0,6	0,6	0,4	0,6	0,4	0,4	0,4	0,6
0,2	0,2	0,4	0,4	0	0,6	0,2	0,2
0,4	0,2	0,6	0	0,6	0,4	0,8	0,4
0,4	0,4	0,2	0,8	0,4	0	0,2	0,4
0,4	0,8	0,6	0,6	0,4	0	0,8	0,6
0,2	0,2	0,6	0,8	0,4	0,2	0,4	0
0,6	0,8	0,4	0,2	0,6	0,4	0,6	0,4
0,2	0,6	0,4	0,2	0,6	0,4	0,4	0,2
0	0,4	0,2	0,2	0,6	0,4	0,4	0
0,4	0,4	0	0,4	0,4	0,4	0,6	0,4
0,4	0,2	0,4	0,2	0	0,4	0,4	0,8
0,8	0	0,8	0,4	0	0,2	0,6	0,4
0,4	0,4	0,4	0,4	0,4	0,4	0,6	0,2
0,2	0,4	0,4	0,4	0,2	0,4	0,2	0,4
0,6	0,2	0,6	0,4	0,4	0,6	0,8	0,6
0,4	0,2	0,2	0,4	0,6	0,4	0,8	0,6
0,4	0	0,6	0,4	0,4	0,2	0,2	0,8
0,6	0,4	0,2	0,2	0,4	0,2	0,4	0,2
0,4	0,2	0,4	0,2	0,4	0,2	0,2	0,4
0,4	0,2	0,2	0,4	0,4	0,4	0,6	0,6
0,6	0,4	0,2	0,8	0,2	0,4	0	0,8

Trial36	Trial37	Trial38	Trial39	Trial40	Trial41	Trial42	Trial43	Trial44
0,2	0,2	0,8	0,6	0,6	0,2	0,4	0,6	0,4
0,6	0,4	0,2	0,4	0,4	0,4	1	0,2	0,4
0,4	0,4	0,4	0,6	0,2	0,2	0,8	0,2	0,4
0	0,6	0,2	0,2	0,6	0	1	0,6	0,6
0,6	0,2	0,8	0	0,2	0,2	0,4	0,8	0
0,6	0,2	0,6	0,2	0,2	0,4	0,2	0,8	0,6
0,4	0,6	0,8	0,4	0,4	0,4	0,8	0,4	0,4
0,8	0,4	0	0,6	0,2	0,4	0,2	0,6	0
0,8	0,6	0,4	0,8	0,4	0,4	0,8	0,6	0,6
0,4	0,4	0,2	0,6	0,4	0,6	0,4	0,6	0,4
0,4	0,6	0,2	0	0,4	0,4	0	0,2	0,4
0,4	0,2	0,4	0	0,4	0,6	0	0,6	0,4
0,6	0,8	0,6	0,2	0,4	0,2	0,6	0,8	0,6
0,4	0,2	0	0	0,2	0,4	0,6	0,2	0,6
0,4	0,4	0,2	0,6	0,2	0,6	0,4	0,2	0,8
0,2	0,4	0,2	0,2	0,4	0,2	0,2	0,4	0,2
0,4	0,2	0,8	0,6	0,4	1	0,2	0,8	0,2
0,8	0,6	0,8	0,4	0,4	0,4	0,8	0,6	0,6
0,4	0,4	0,8	1	0,8	0,4	0,6	0,4	0,4
0,6	0,6	0,4	0,4	0,2	0,2	0,4	0,6	0,2
0,6	0,8	0	0,6	0,8	0,6	0,6	0,2	0,2
0,4	0,4	0,4	0,2	0,4	0,2	0,2	0,6	0
0,8	0,6	0,4	0,2	0,2	0,4	0,6	0,4	0,6
0,6	0,4	0,2	0,4	0,4	0,4	0,8	0,6	0,6
0,8	0,6	0,6	0,6	0,4	0,2	0,4	0,6	0,6
0,4	0,2	0,6	0,4	0,2	0,4	0	0,4	0,2
0,6	0	0,6	0,6	0,4	0,2	0,6	0,8	0,4
0,4	0,4	0,6	0,6	0,4	0,6	0,4	0,4	0,2
0,6	0,4	0,6	0,4	0,2	0,2	0,2	0,6	0,2
0,4	0,6	0,4	0,4	0,2	0,4	0,6	0,4	0,4
0	0	0,6	0,6	0,6	0,2	0,2	0,4	0
0,4	0,8	0,8	0,2	0,4	0,2	0,8	0,2	0,6
0,6	0,6	0,6	0,4	0,2	0,4	0,4	0,2	0,4
0,4	0,6	0,4	0,4	0,6	0,4	0,6	0,4	0,6
0,6	0,4	0,2	0,4	0,4	0,2	0,4	0,6	0
0,4	0,2	0,2	0,6	0,4	0,4	0,6	0,2	0,6
0	0,6	0,2	0,2	0,6	0,2	0,4	0	0,6
0,6	0	0,2	0,4	0,4	0,2	0,2	0,8	0,2
0,4	0,2	0,6	0	0,4	0,8	0,2	0,4	0,2
0,4	0	0,6	0	0,2	0,6	0,4	0,4	0,6
0,4	0,6	0,4	0,8	0	0,2	0,6	1	0,4
0,4	0,8	0,8	0,6	0,4	0,4	0,8	0,4	0,2
0,2	0,2	0,6	0,4	0,6	0,2	0,4	0,6	0,6
0,4	0,6	0,4	0,4	0,6	0,2	0,4	0,4	0,6
0,2	0,2	0,4	0,6	0,2	0,8	0,4	0,6	0,6
0,6	0,8	0,4	0,4	0,2	0,4	0,4	0,2	0,2
0,4	0,6	0,2	0,8	0,2	0,2	0,4	0,2	0,2
0,4	0,4	0,4	0,4	0	0,2	0,4	0,4	0,4
0,6	0,2	0,2	0,6	0,4	0,4	0,8	0,2	0,4
0	0,2	0,2	0,2	0,2	0,2	0,2	0,2	0,2
0,2	0,4	0,4	0	0,8	0,4	0,8	0,6	0,6
0,6	0,2	0,6	0,4	0,2	0,4	0,4	0,4	0

Trial45	Trial46	Trial47	Trial48	Trial49	Trial50	Trial51
0,8	0,2	0,6	0,6	0,6	0,2	0,4
0,4	0,8	0,2	0,8	0,6	0,6	0,2
0,2	0,8	0,6	0,4	0,2	0,4	0,6
0,6	0,6	0,2	0,6	0,6	0,4	0,4
0,4	0,4	0,6	0,6	0,6	0,8	0,4
0,6	0	0,4	0,6	0,2	0,8	0,4
0,4	0,4	0,6	0,2	0,4	0,2	1
0,6	0,4	1	0,4	0,4	0,6	0,8
0,6	0	0,8	0,6	0,4	0,6	0,8
0,6	0,2	0,6	0,6	0,4	0,4	0,4
0	0,2	0,4	0	0,8	0,4	0,2
0,2	0,6	0,4	0,8	0,4	0,6	0,6
0,6	0,4	0,2	0,4	0,4	0,2	0,2
0,4	0	0,2	0,6	0,4	0,2	0,2
0,6	0,6	0,6	0,6	0,4	0,8	1
0,4	0,4	0	0	0,4	0,4	0,2
0,6	0,6	0,6	0,4	0,8	0,2	0,6
0,2	0,2	0,6	0,2	0,4	0,8	0,4
0,4	0,6	0,6	0,6	0,6	0,2	0,8
0,6	0,2	0,8	0,2	0,6	0,2	0,6
0,4	0,8	0,8	0,6	0,6	0,2	0,6
0,6	0	0,6	0,4	0,4	0,4	0,2
0,6	0,6	0,6	0,2	0,8	0,4	0,8
0,2	0,6	0,2	0,4	0,6	0,4	0,4
0,6	0,4	0,2	0,8	0,2	0,4	0,4
0,2	0	0,6	0,6	0,4	0,6	0,4
0,8	0,2	0	0,6	0,4	0,4	0,4
0,6	0,4	0,6	0,4	0,4	0,6	0,4
0,2	0,6	0,4	0,2	0,6	0,6	0
0,2	0,6	0,4	0,4	0,6	0,4	0,6
0,6	0,2	0,2	0,6	0,2	0,4	0,4
0,2	0,6	0,4	0,6	0,6	0,4	0,4
0,6	0,2	0,8	0	0,2	0,2	0,4
0,4	0,8	0,6	0,4	0,6	0,6	0,4
0	0,2	0,6	0,4	0,6	0,2	0,4
0	0,8	0,4	0,8	0,2	0,2	0,4
0,4	0,4	0,4	0,4	0,6	0,4	0,4
0,6	0,2	0,4	0	0	0,8	0,2
0,8	0,4	1	0,6	0,4	0,4	0,2
0,6	0,6	0,6	0,6	1	0	0,2
0,6	0,4	0,6	0,2	0,8	0,6	0,6
0,6	0,8	0,2	0,6	0,4	0,4	0,4
0	0,4	0,6	0,2	0,6	0,2	0,4
0,6	0,4	0,6	0,6	0,4	0,8	0,2
0,4	0,6	0	0,4	0,6	0	0,2
0,4	0	0,6	0	0,6	0,4	0,2
0,4	0,6	0,8	0,2	0,6	0,6	0,4
0,8	0,4	0,4	0,2	0	0,4	0,8
0,4	0,4	0,4	0,2	0,2	0,4	0,4
0,4	0,6	0,2	0,4	0,4	0,6	0,2
0,2	0,6	0,4	0,8	0,4	0,4	0,2
0,6	0,2	0,2	0,6	0,4	0,6	0,2

Trial52	Trial53	Trial54	Trial55	Trial56	Trial57	Trial58	Trial59	Trial60	Trial61	Trial62	Trial63	Trial64	Trial65	Trial66	Trial67	Trial68	Trial69	Trial70	Trial71	Trial72	Trial73	Trial74	Trial75	Trial76	Trial77	Trial78	Trial79	Trial80	Trial81	Trial82	Trial83	Trial84	Trial85	Trial86	Trial87	Trial88	Trial89	Trial90	Trial91	Trial92	Trial93	Trial94	Trial95	Trial96	Trial97	Trial98	Trial99	Trial100
0,4	0,4	0,2	0,6	0,4	0,6	0,6	0,4	0,4	0	0,6	0,4	0,8	0,6	0,6	0,6	0,4	0,4	0,4	0,2	0,6	0,6	0,4	0,6	0,2	0,6	0,6	0,4	0,4	0,4	0,6	0,4	0,6	0,6	0,4	0,6	0,8	0,2	0,8	0,6	0,8	0,2	0,8	0,4	0,6	0,4	0,6	0,6	0,4
1	0,4	0,6	0,2	0,2	0,6	0,6	0,4	0,6	0,6	0,2	0,2	0,4	0,8	0,6	0,4	0,4	0,8	0,4	0	0,6	0,6	0,2	0,4	0,4	0,8	0,4	0,6	0	0,6	0,8	0,4	0,8	0,6	0,6	0,8	0,4	0,4	0,4	0,2	0,2	0,6	0,2	0,8	0,8	0,6	0,6	0,4	0,6
0,6	0,4	0,4	0,6	0,6	0,8	0,2	0,6	0	0,2	0,2	0,6	0,8	0,4	0,6	0,2	0,2	0,2	0,8	0,4	0,4	0,2	0,8	0,4	0,4	0,2	0,2	0,6	0,4	0,2	0,8	0,2	0,8	0,4	0,2	0,6	0,8	0,4	0,2	0,6	0,6	0,4	1	0	0,2	0,4	1	0,6	0,4
0,8	0,6	0,4	0,4	0,8	0,2	0,4	0,6	0,6	0,2	0,6	0,6	0,6	0,2	0,6	0,4	0,4	0,4	0,8	0,2	0,8	0	0,2	0,8	0,6	1	0,6	0,6	0,4	0,4	0,8	0,6	0,6	0,2	0,6	0,6	0,6	0,6	0,2	0,6	0,6	0,6	0,8	0,4	0,4	0,6	0,4	0,2	0,4
0,8	0,8	0,2	0,6	0,2	0,6	0,6	0,4	0,4	0,2	0,2	0,6	0,6	0,6	0,2	0,4	0,6	0,8	0,2	0,4	0,4	0,4	0,2	0,6	0,6	0,4	0,8	0,4	0,4	0,2	0,4	0,8	0,6	0,6	0,2	0,6	0,6	0,6	0,4	0,4	0	0,4	0,8	0,4	0,2	0,6	0,6	0,8	0,6
0,4	0,4	0,2	0,4	0,4	0,4	0,6	0,6	0,6	1	0,8	0,6	0,4	0,8	0,8	0,6	0,8	0,6	0,4	0,8	0,2	0,6	0,4	0,8	0,4	0,6	0,4	0,2	0,8	0,6	0,6	0,4	0,6	0,6	0,4	0,6	1	0	0,4	0,4	0,6	1	0,4	0,6	0,2	0,4	0	1	0,6
0,6	0,6	0,6	0,4	0,2	0,2	0,4	0,4	0,2	0,6	0,2	0,2	0,4	0,6	0	0,4	0,4	0,4	0	1	0,4	0,4	0,4	0,2	0,4	0,6	0,6	0,4	0,6	0,6	0,4	0,4	0,4	0,6	0,4	0,6	0	0,8	0,4	0,4	0,2	0,6	0,6	0,2	0,8	0,2	0,2	0,6	0,6
0,8	0,4	0,4	0,4	0,6	0,8	0,8	0,4	0,6	0,2	0,6	0,8	0,4	0,8	0,8	0,6	0,8	0,4	1	0,2	0,4	0,4	0,4	0,8	0,2	0,8	0,4	0,6	0,6	0,2	0,8	0,4	0,6	0,4	0,8	0,6	1	0,6	0,4	0,6	0	0,4	0,8	0,4	0,8	0,8	0,8	0,2	0,4
0,6	0,4	0,2	0,2	0,6	0,2	0,4	0,4	0,6	0,6	0,2	0,2	0,6	0,2	0,6	0,4	0,2	0,4	0,4	0,6	0,6	0,4	0,4	0,8	0,4	0,6	0,8	0,6	1	0,2	0,8	0,4	0,4	0,8	0,6	1	0,4	0,4	0,2	0,6	0,4	0,8	0,2	0,2	0,4	0,4	0,2	0,8	0,4
0,6	0	0,4	0,4	0,4	0,4	0,6	0,4	0,8	0,2	1	0,4	0,6	0,6	0,6	0,6	0,2	0,8	0,2	0,6	0,6	0,4	1	0	0,8	0,4	0,8	0,2	0,6	0,6	0,4	0,4	0,2	0,6	0,6	0,4	0,6	0,6	0,6	0,4	0,6	0,6	0,4	0,8	0,2	0,6	1	0,4	0,6
0,6	0,6	0,2	0,4	0	0,6	0,2	0,4	0,4	0,4	0,4	1	0,6	0,4	0,4	1	0	0,2	0,2	0,6	0,2	0	0,6	0,2	0,4	0,6	0,4	0,8	0,2	0,8	0,8	0,2	0,4	0,4	0,2	0,6	0	0,8	0,2	0,6	0,6	0	0,8	0,4	0,2	0,4	0,6	0,2	0,4
0,4	0,6	0,2	0,6	0,2	0,2	0,4	0,6	0,2	0,8	0,2	0,4	0,4	0,8	0,4	0,6	0,8	0,4	0	0,4	0,2	0,4	0,6	0,4	0,6	0,4	0,6	0,4	0,8	0,6	0,6	0,2	0,8	0,2	0,6	1	0,4	0,8	0,6	0,8	0,6	0,4	0,2	0,6	0,2	0,4	0,2	0,4	0,6
0,4	0,8	0,6	0,4	0,8	1	0,4	0,4	0,4	0,2	1	0,6	0,6	0,4	0,6	1	0,4	0,8	0,6	0,8	0,6	0,4	0,4	0,4	0,4	0,4	0,2	0,4	0,8	0,2	0,6	0,4	0,8	0,6	0,4	0	0,6	0,2	0,4	0,2	0,6	0,4	0,4	0,6	0,6	0,2	0,2	0,4	0,2
0,6	0,6	0,4	0,4	0,4	0,6	0,4	0	0,4	0,2	0,4	0,2	0,2	0,6	0,2	0,4	0,4	0,2	0,2	0,4	0,8	0,4	0,6	0,2	0,6	0,2	0,6	0,4	0,8	0,8	0,2	0,2	0,4	0	0,4	0,6	0,4	0,4	0,4	0,6	0,2	0,4	0,6	0,6	0,4	0,2	0,4	0,6	0,6
0,4	0,2	0,4	0,6	0,4	0,8	0,4	0,6	0,4	0,8	0,4	0,2	0,6	0,2	0,8	0,4	1	0	0,8	0,4	0,4	0,8	0,6	0,2	0,6	0,4	0,4	0,4	0,8	0,6	0,2	0	0,4	0,6	0,4	0,8	0,4	1	0,2	1	0,4	0,4	0,2	0,4	0,8	0,6	0,2	0,4	0,6
0,2	0,4	0,6	0,8	0,2	0,2	0,8	0,6	0,4	0,6	0,4	0,4	0,4	0,4	0,2	0,2	0,4	0,6	0,4	0,4	0,4	0,4	0,2	1	0,6	0,8	0,4	0	0,4	0,6	0,6	0,4	0,8	0,4	0,8	0,2	0,6	0,6	0,4	0,6	0,4	0,4	0,6	0,6	0,6	0,2	0,4	0,4	0,8
0,6	0,4	0,4	0,6	0,6	0,4	0,8	0,6	0,4	0,4	0,6	0,6	0,4	0,6	0,6	0	0,6	0,6	0,6	0,6	0,4	0,4	0,4	0,6	0,6	1	0,4	0,2	0,8	0,4	0,6	0,6	0,6	0,6	0,6	0,8	0,4	0,4	0,8	0,6	0,8	0	0,6	0,6	0,4	0,4	0,8	0,4	0,8
0,4	0,6	0,4	0,4	0,6	0,8	0,2	0,8	1	0	0,6	0,8	0,6	0,4	0,6	0,6	0,4	0,8	0,4	0,6	0,6	0,6	0,2	0,8	0,6	0,2	0,2	0,6	0,4	0,4	0,4	0,8	0,2	0,4	0,6	0,6	0,6	0,6	0,8	0,4	0,8	0,4	0,4	0,6	0,4	0,6	0,4	0,8	0,2
0,6	0,2	1	0,6	0,4	0,6	0,6	0,6	0,4	0,8	0,6	0,8	0,8	0,4	0,8	0,2	0,2	0,6	0,6	0,8	0,2	0,6	0,4	0,8	0,6	0,4	0,6	0,6	0,6	0,4	0,4	0,6	0,4	0,4	0,6	0,2	0,4	0,8	0,4	0,4	0,4	0,8	0,8	0	1	0,4	0,8	0,4	0,4
0,4	0,4	0	0,6	0,4	0,6	0,6	0,4	0,6	0,2	0,2	0,8	0,4	0,6	0,4	0,4	0,6	0,6	0,4	0,8	0,2	0	0,8	0,8	0,2	0,6	0,4	0,4	0,4	0,4	0,6	0,4	0,2	0,4	0,4	1	0,6	0,4	0,4	0,6	0,6	0	0,8	0,4	0,4	0,2	0,8	0,4	0,4
0,6	0	0,6	0,4	0,8	0,2	0,8	0,6	0,6	0,8	0,6	0,8	0,8	0,8	0,4	1	0,4	0,8	0,4	0,6	0,6	0,6	0,8	0,6	0,8	0,4	0,8	0,2	0,4	0,2	0,6	0,8	0,2	0,8	0,2	0,6	0,6	0,6	0,6	0,6	0,4	0,2	0,8	0	0,6	0,6	1	0,2	0,8
0,8	0,6	0,2	0,6	0,2	0,6	0,2	0,6	0,2	0,4	0,6	0,6	0,4	0,6	0,6	0,4	0,6	0,6	0,6	0,4	0,6	0,8	0,8	0,4	0,4	0,6	0,4	0,2	0,4	0,2	0,6	0	0,6	0,6	0,4	0,2	0,2	0,2	0,6	0,2	0,8	0,4	0,8	0,2	0,4	0,4	0,6	0,2	0,2
0,4	0,2	0	0,6	0,2	0,8	0,4	0,6	0,6	0,6	0,2	0,6	0,6	0,4	0,4	0,6	0,4	0,8	0	1	0,6	0,4	0,8	0,2	0,4	0,8	0,2	0,6	0,4	0,4	0,4	0,6	0,8	0,4	0,4	0,2	0,8	0,4	0,4	0,4	0,4	1	0,4	0,6	0,4	0,2	1	0,6	0,2
0,4	0,4	0,8	0,4	0,6	0,4	0,4	0,6	0,6	0,2	0,2	0,4	0,4	0,8	0,4	0,4	0,4	0,2	0,6	0,6	0,2	0,4	0,6	0,4	0,2	0,4	0,4	0,4	0,4	0,6	0,6	0,4	0,8	0,4	0,8	0,6	0,6	0,8	0,6	0,4	0,8	0,4	0,6	0,6	0,4	0,6	0,6	1	0,2
0,4	0,2	0,2	0,4	0,4	0,4	0,6	0,2	0,8	0,4	0,6	0,2	0,6	0,4	0	0,4	0,2	0,4	0,4	0,6	0,4	0,2	0,4	0,4	0,6	0,4	0,2	0,6	0,6	0,4	0,6	0,4	0,4	0,6	0	0,2	0,4	0,2	0,8	0,4	0,6	0,4	0,6	0,6	0,4	0,6	0,4	0,4	0,4
0,6	0,6	0,4	0,4	0,2	0,4	0,6	0	0,8	0	0,8	0,4	0,2	0,4	0,6	0,6	0,6	0,4	0,4	0,4	0,6	0,8	0,8	0,8	0,8	0,4	0,8	0,2	0,6	0,6	0,4	0,4	0,6	0,8	0,6	0,6	0,2	0,4	0,4	0,4	0,2	0,8	0,4	0,8	0,6	0,8	0,6	0,6	0,6
0,4	0,4	0,4	0,6	0,4	0,6	0,4	0,8	0	0,6	0,2	0,8	0,6	1	0,2	0,8	0,4	0,4	0,6	0,8	0,8	0,2	0,8	0	0,4	0,4	0,2	0,4	0,6	0,8	0,6	0,2	0,2	0,2	0,8	0,2	0,4	0,8	0,6	0,4	0,6	0,8	0,8	0,4	0,6	0,8	0,6	0,4	0,8
0	0,6	0,6	0,2	0,4	0,6	0,4	0,4	0,8	0,2	0,4	0,6	0,8	0,6	0,6	0,8	0,4	0,4	0,8	0,4	0,4	0,6	0,8	0,6	0,4	0,4	0,8	0,2	0,6	0,2	0,4	0,6	0,2	0,8	0,4	0,4	0,6	0,4	0,4	0,2	0,6	0,4	0,6	0,6	0,4	0,8	0,4	0,2	0,4
0,2	0,4	0,6	0,6	0,2	0,4	0,4	0,4	0,6	0,2	0,2	0,6	0,4	0,6	0,2	0,6	0,4	0,8	0,8	0,6	0,4	0,2	0,4	0,2	0,6	0,4	0,6	0	0,6	0,4	0,4	0,4	0,8	0,2	0,6	0,2	0,6	0,8	0,6	0,2	0	0,4	0,4	0,8	0,4	0,4	0,4	0,4	0,4
0,2	0,4	0,4	0,2	0,6	0,6	0,4	0,6	0,6	0,4	0,4	0	0,4	0,8	0,4	0,6	0,4	0,2	0,4	0,6	0,4	0,2	0,6	0,6	0,6	0,6	0,6	0,4	0,6	0,8	0	0,2	0,6	0,2	0,8	0,6	0,6	0,8	0,6	0	0,4	0,4	0,6	0,6	0,2	0,6	0,8	0,8	0,2
0	0,6	0,2	0,4	0,2	0,4	0,4	0,2	0,4	0,2	0,4	0,6	0,2	0,2	0,2	0,4	0,2	0,2	0,6	0,2	0,4	0,2	0,4	0	0,2	0,4	0,2	0,8	0,8	0,4	0,4	0,4	0	0,8	0,4	0,2	0,2	0,4	0,4	0,6	0,2	0,6	0,6	0,4	0,6	0,4	0,4	0,6	0,2
0,2	0,6	0,4	0,2	0,4	0,6	0,8	0,2	0,4	0,4	0,6	0,2	0,6	0,4	0,4	0,4	0,4	0,8	0,2	0,4	0,4	0,2	0,2	0,8	0,4	0,8	0,6	0,8	0,4	0,8	1	0,6	0,2	0,6	0,6	0,6	0,8	0,4	0,6	0,6	0,8	0,4	0,6	0,6	0,6	0,6	0,4	0,6	0,6
0,4	0,4	0,4	0,6	0,8	0,4	0	0,4	0,4	0,6	0,4	0,4	0,8	0,6	0,2	0,2	0,2	0,4	0,2	0,4	0,2	0,4	0,6	0,6	0,4	0,2	0,4	0,2	0,4	0,8	0,2	0,6	0,6	0,6	0,2	0,4	0,4	0,4	0,4	0,6	0,4	0,4	0,4	0,8	0,2	0,6	0	0,6	0,6
0,6	0,4	0,8	0,4	0,4	0,6	0,2	0,4	0,2	0,2	0,2	0,6	0,6	1	0	0,8	0,6	0,6	0,6	0,4	0,2	0,4	0,4	0,6	0,8	0,8	0,8	0,8	0,4	0,6	0,4	0,8	0,4	0	0,4	0,6	0,4	0,4	0,2	0,4	1	0,8	0,4	0,4	0,4	1	0,8	0,2	0,6
0,4	0,4	0,4	0,4	0,4	0,2	1	0	0,6	0,6	0,6	0,2	0,6	0,8	0	0,6	0,4	0,6	0,8	0,4	1	0,6	0,4	0,2	1	0,4	0,4	0,2	0,4	0,4	0,4	0,8	0,6	0,6	0,2	0,6	0,2	0,4	0,6	0,4	0,6	0,4	1	0,4	0,4	0,6	0	0,4	0,2
0	0,4	0,8	0,4	0,8	0,2	0,6	0,2	0,4	0,6	0,6	0,6	0,6	0,2	0,2	0,6	0,6	0,4	0,4	0,6	0,8	0,2	0,6	0,6	0,6	0,6	0,6	0,6	0,6	0,2	0,2	0,6	0,8	0,4	0,6	0,4	0,4	0,2	0,8	0,4	0,8	0,2	0,4	0,6	0,2	0,4	0,6	0,6	0,4
0,4	0,2	0,6	0,6	0,6	0	0,8	0,4	0,4	0,2	0,2	0,4	0,4	0,6	1	0,6	0,2	0,6	0,6	0,8	0,2	0,6	0,4	0,4	0,4	0,6	0,2	0,8	0,2	0,4	0,6	0,2	0,6	0	0,4	0,4	0,4	0,6	0,8	0,6	0,6	0,2	0,4	0,4	0,6	0,6	0,4	0,4	0,2
0,2	0,2	0,2	0,6	0,4	0,2	0,4	0,6	0,4	0,4	0,6	0,8	0,6	0,2	0	0,2	0,6	0,2	0,2	0,6	0,6	0,2	0,4	0,4	0	0,4	0,4	1	0,4	0	0,2	0,6	0,2	0,6	0	0,2	0,8	0,6	0,4	0,6	0,8	0,6	0,4	0,6	0,4	0,6	0,4	0,4	0,6
0,6	0,4	0,2	0,6	0,4	0,4	0,4	0,4	0,4	0,4	0,4	0,4	0,4	0,4	0,4	0,4	0,6	0,4	0,6	0,4	0,8	0,4	0,4	0,6	0,8	0,6	0,4	0	0,8	0,6	0,4	0,6	0,8	0,6	0,4	0,4	0	0,4	0,6	0,2	0,4	0,6	0,8	0,6	0,4	0,8	0,4	0,8	0,4
0,4	0,4	0,6	0,4	0,2	0,4	0,8	0,8	0,2	0,2	0,2	0,8	1	0,8	0,4	0,4	0,4	0,6	0,4	0,4	0,4	0,4	0,6	0,6	0,4	0,4	0,2	0,4	0,6	0,2	1	0,8	0,2	0,4	0	1	0,4	0,6	0,6	0,4	0,4	0,4	0,8	0,8	0,6	0,4	0,6	0,4	0,4
1	0,4	0,4	0,6	0,6	0,6	0,6	1	0,8	0,6	0,8	0,4	0,6	0,4	0,6	0,6	0	0,6	0,4	0,4	0,6	0,6	0,4	0,4	0,6	0,2	0,6	0,8	0,4	0,6	0,4	0,4	1	0,6	1	0,2	0,8	0,2	0	0,6	0,4	1	0,4	0,4	0,8	0,4	0,4	0,6	0
0,6	0,4	0,2	0,4	0,4	0,4	0,6	0,2	0,2	0	0,4	0,2	0	0,2	0,4	0,2	0,6	0,2	0,6	0,6	0,2	0,6	0,6	0,4	0	0,6	0,8	0,2	0,6	0,4	0,4	0,4	0,4	0,4	0,4	0,2	0,4	0,8	0,6	0,6	0,4	0,6	0,8	0,6	0,4	0,6	1	0,4	1
0,2	0,6	0,6	0,4	0,4	0,4	0	0,4	0,4	0,2	0,2	0,6	0,4	0,8	0,8	0,2	0,8	0,4	0,2	0,6	0,4	0,6	0,4	0,8	0,8	0,6	0,4	0,6	0,6	0,6	0,6	0,2	0,4	0,6	0,2	0,4	0,4	0,4	0,8	0,8	1	0,2	0,8	0,6	0,4	0,6	0,6	0,2	0,4
0,4	0,6	0,6	0	0,8	0,4	0,4	0,6	0,4	0,2	0,2	0,2	0,8	0,4	0,6	0,6	0,6	0,4	0,4	0,6	0,6	0,6	0,6	0,4	0,4	0,6	0,6	0,6	0,8	0,4	0,2	0,6	0,4	0,2	0,2	0,4	0,6	0,8	0,4	0,6	0,2	0,6	0,4	0,4	0,8	0,2	1	0,8	0,4
0,4	0,6	0	0,4	0	0,2	0,2	0,8	0,6	0,6	0,6	0,4	0,2	0,4	0,2	0,8	0,2	0,4	0,4	0,8	0,4	0,6	0,4	0,2	0,6	0,8	0,2	0,2	0,4	0,8	0,4	0,4	0,2	0,4	0,8	0,6	0,2	0,6	0,6	0,6	0,6	0,8	0,6	0,2	0	0,4	0,4	0,2	0,2
0,4	0,6	0,6	0,2	0,6	0,4	0,4	0,6	0,8	0,6	0,4	1	0,4	0,4	0,8	0,4	0,6	0,2	0,8	0,2	0,6	0,8	0,6	0,8	0,4	0,6	0,6	0,2	0,4	0,2	0,4	1	0,6	0,2	0,6	0,4	1	0,2	0,2	0,8	0,8	0,6	0,6	0,6	0,2	0,6	0,4	0,6	0,8
0,6	0,4	0,4	0,8	0,4	0,6	0,4	0,2	0,6	0,4	0,2	0,8	0,4	1	0,4	0,6	1	0,4	0,6	0	0,6	0,6	0,2	0,4	0,4	0,6	0,4	0,4	0,8	0,2	0,2	0,2	0,2	0,8	0,8	0,6	0,4	0,6	0,2	0,6	0,4	0,6	0,2	0,2	0,2	0,2	0,4	0,2	0,6
0,2	0,4	0	0,6	0,4	0,4	0,2	0,6	0,4	0,2	0,6	0,8	0,6	0,6	0,4	0,2	0,6	0,8	0,4	1	0,4	0,6	0,2	0,6	0,4	0,4	0,4	0,6	0	0,8	0,2	0,6	0,6	0	0,6	0,4	0	0,4	0,6	0,2	0,4	0,4	0,4	0,6	0,4	0,4	0,6	0,4	0,8
0,6	0,4	1	0,8	0,8	0,2	0,8	0,4	0,4	0,6	0,2	0,8	0,2	0,8	0,4	0,4	0	0,4	0,6	0,4	0,4	0,2	0,6	0,6	0,4	0,4	0,6	0,2	0,6	0,2	0,2	0,4	0	0,6	0,6	0,4	0,4	0,6	0,8	0,6	0	0,6	0,2	0,4	0,6	0,2	0,8	0,6	0,4
0,2	0,4	0,4	0,6	0,6	0,2	0,6	0	0,4	0,2	0,4	0,2	0	0,6	0,4	0,2	0,2	0,4	0,4	0,6	0,2	0	0,2	0,6	0,4	0,6	0,2	0,2	0,4	0,4	0,8	0,2	0,4	0,4	0,2	0,2	0,4	0,4	0,6	0,4	0,6	0,8	0,4	0,2	0,6	0,4	0,6	0,2	0,6
0,4	0,6	0,4	0,6	0,8	0,2	0,4	0,2	0,8	0,4	0	1	0,4	0,2	0,8	0,6	1	0,4	0,4	0,6	0,6	0,6	0,4	0,4	0,6	0,4	0,4	0,6	0,6	0,6	0,6	0,2	0,6	0,2	0,6	0,2	0,8	0,6	0,4	0,8	0,4	0,4	1	0,8	0,2	0,8	0,2	0,6	0,2
0,6	0,2	0,8	0,2	0,6	0,2	0,4	0,2	0,6	0	0,2	0,4	0,8	0,6	0,6	0,6	0,4	0,8	0,6	0,2	0,6	0,4	0,4	0,4	0,8	0,8	0,6	0,2	0,4	0,8	0,4	0,6	0,6	0,6	0,4	0,4	0,2	0,2	0,2	0,6	0,4	0,6	0,4	0,6	0,4	0,4	0	0,6	0,4
